# The Pharmacokinetic Properties of Veterinary Antiparasitic Drugs in the Context of Human Pregnancy—An In Silico Study

**DOI:** 10.3390/ijms27135921

**Published:** 2026-06-30

**Authors:** Anna W. Sobańska, Ewa Okoń, Andrzej M. Sobański

**Affiliations:** 1 Department of Analytical Chemistry, Medical University of Lodz, 90-151 Łódź, Poland; ewa.okon@stud.umed.lodz.pl; 2 Faculty of Chemistry, University of Lodz, 91-403 Łódź, Poland; andrzej.sobanski@edu.uni.lodz.pl

**Keywords:** antiparasitic veterinary drugs, acetylcholinesterase inhibitors, placenta permeability, QSAR

## Abstract

In this study, 86 veterinary antiparasitic drugs were investigated for their ability to cross the human placenta, expressed as the Clearance Index (***CI***) relative to antipyrine. It was established that the ***CI*** is positively correlated with the permeability of compounds through Caco-2 cells (***caco2***) and with ***ATSC7i***—the centered Broto-Moreau autocorrelation—lag 7/weighted by the first ionization potential, and inversely correlated with Topological Polar Surface Area (***TPSA***_Mord_). All the studied drugs might cross the placenta, some rapidly, as suggested by their ***CI*** values. For some of the antiparasitic drugs (especially those from the pyrethroid family) investigated in this study, the placental permeability predicted using the ***CI*** values differed from predictions based on the equilibrium fetus-to-mother concentration ratio (***FM***), indicating the possibility of rapid clearance or metabolism. According to our QSAR analysis, almost all of the studied drugs have AChE inhibition constants (***K***_i_) comparable to those of AChE inhibitors registered in the CHEMBL database. Molecular docking studies revealed that the drugs might engage in several types of bonds/interactions, mainly with tryptophan (Trp86, Trp286), tyrosine (Tyr124, Tyr227, Tyr341), and phenylalanine (Phe295, Phe297, and Phe338)—and with at least one amino acid from the AChE catalytic triad.

## 1. Introduction

The optimal development of a fetus largely depends on the placental transfer of gases, hormones, and nutrients from the maternal to the fetal compartment, and of waste products in the opposite direction. Unfortunately, unwanted xenobiotics cross the placenta via the same mechanisms as nutrients. Hence, almost all drugs and most environmental contaminants cross the placenta to some extent. Some undesired compounds accumulate in the placenta and interfere with its functions, thereby affecting fetal development, despite their poor placental permeability [1]. The placenta is not only a barrier; it also produces various hormones, cytokines, and growth factors that support fetal development. It contains influx and efflux transporters from two major superfamilies, adenosine triphosphate-binding cassette (ABC) and solute carrier (SLC), and several enzymes including Phase I and Phase II xenobiotic-metabolizing enzymes similar to those found in the liver [2,3,4,5,6].

Acetylcholinesterase is a key enzyme in the nervous system, primarily known for its ability to hydrolyze the neurotransmitter acetylcholine (ACh) at rates limited only by the diffusion of ACh to the active site of the enzyme [7]. Other studies have revealed that AChE plays several roles in human and animal physiology; for example, it is an important factor in neurodegeneration [8]. Acetylcholine and acetylcholinesterase are present in human epithelial cells in the airways, alimentary tract, urogenital tracts, and epidermis, as well as in mesothelial cells and endothelial, muscle, and immune cells [9]. Non-neuronal acetylcholine, cholinesterase, and receptors (muscarinic and nicotinic) act as a local cellular signaling system, which has also been identified in the placenta [10,11].

Acetylcholinesterase inhibitors are structurally diverse compounds that block AChE activity, either reversibly or irreversibly [12]. Reversible inhibition of AChE is usually associated with therapeutic effects, whereas irreversible inhibition is associated with toxicity; however, the reality is more complex. Some reversible AChE inhibitors (e.g., donepezil, galantamine, and rivastigmine) improve brain function in patients with mild-to-moderate cognitive disorders (including Alzheimer’s disease). In dementia with cholinergic deficits, cognitive function improves by increasing acetylcholine levels at synapses. Reversible AChE inhibitors are also used to treat myasthenia gravis, postoperative ileus, bladder distention, glaucoma, and anticholinergic overdose [13]. Irreversible AChE inhibitors are generally toxic owing to sustained cholinergic overstimulation and are typically used as nerve agents (tabun, sarin, soman, and VX) or insecticides (illegal in some countries and often restricted to particular applications, e.g., tetrachlorvinphos and malathion). However, some compounds from this group (diisopropyl fluorophosphate, echothiopate) are used to treat glaucoma [13].

Veterinary antiparasitic drugs act against target organisms (see Supplementary Materials) via several mechanisms:

- Pyrethroids: interactions with sodium channels and the induction of prolonged depolarization in neurons. Compounds from this family are more than three orders of magnitude more toxic to insects (e.g., fleas and lice) than to mammals and are considered relatively safe for pregnant females and offspring when used externally [14]
- Benzimidazoles: β-tubulin binding and inhibition of microtubule polymerization.
- Macrocyclic lactones (ivermectin, moxidectin): interactions with glutamate-gated chloride channels.
- Phenylpyrazoles, neonicotinoids, isoxazolines, and spinosyns: interactions with target-specific ion channels or receptors.
- Insect growth regulators: disruption of the juvenile hormone or chitin synthesis.
- Quinoline derivatives, nitroimidazoles, and triazines: suppression of acid synthesis or unique metabolic pathways.

An important mechanism of antiparasitic activity is the inhibition of AChE, which leads to acetylcholine accumulation, overstimulation of cholinergic receptors, paralysis, and death of the target invertebrate. Veterinary antiparasitic drugs that interfere with the cholinergic systems of target and off-target organisms belong to different chemical families:

- Organophosphates: irreversible phosphorylation of the serine residue in the AChE active site, leading to enzyme inactivation and persistent cholinergic toxicity [15,16].
- Carbamates: reversible carbamylation of the AChE active site (serine hydroxyl) [15,17].
- Drugs such as morantel or pyrantel are nicotinic acetylcholine receptor agonists [18].

To date, relatively little attention has been paid to the effects of fetal exposure to veterinary antiparasitic drugs. It remains unclear which drugs from this group are fetotoxic, and what the mechanisms of possible fetotoxicity are, although it is suspected that the interactions of some drugs with the placental or fetal cholinergic systems [19] can be risk factors. The effects of maternal exposure to irreversible AChE organophosphate inhibitors are well recognized [20,21,22], and reversible AChE inhibitors used to treat conditions such as myasthenia gravis in humans (e.g., pyridostigmine) are considered safe with appropriate management [23]. There are insufficient data on the safety of other AChE inhibitors during pregnancy.

The consequences of uncontrolled fetal exposure to AChE inhibitors include long-term neurodevelopmental deficits [24,25,26,27]. This risk is particularly high for irreversible organophosphate AChE inhibitors, such as chlorpyrifos (restricted globally but still used in some countries, e.g., to protect cattle against ectoparasites). It was established that “prenatal exposure to pesticides-at levels not producing adverse health outcomes in the mother-can cause lasting adverse effects on brain development in children” [24]. Animal studies have shown reduced fetal cerebral cortical cholinesterase activity and elevated stillbirth and neonatal death rates in offspring of animals exposed to isoflurane, parathion, and methyl parathion [28], as well as developmental neurotoxicity of carbamates [29]. The placental cholinergic system can also be disrupted by organochlorine compounds (e.g., pesticides such as DDT and DDE), mainly through increased expression of muscarinic receptors [30], with potential consequences including pregnancy complications and impaired fetal development. AChE inhibitors from other chemical families may alter brain morphogenesis, likely through mechanisms unrelated to acetylcholine hydrolysis [31].

Novel acetylcholinesterase inhibitors have recently attracted considerable attention. The ability of chemicals to inhibit AChE has been studied using animal models, as well as in vitro and in silico tools, in the search for new treatments for myasthenia gravis, Parkinson’s disease, Huntington’s disease, schizophrenia, glaucoma, multiple sclerosis, and nerve agent poisoning [32]; however, the main focus of these investigations is on Alzheimer’s disease. Tacrine (discontinued in many countries due to severe side effects) is the oldest drug thought to improve the condition of Alzheimer’s patients based on the cholinergic hypothesis [33]. Since it was patented for Alzheimer’s treatment, several computational studies of AChE inhibitors have been reported for compounds from various chemical families: tacrine/acridine derivatives [34,35,36,37,38,39]; tertiary amine derivatives of cinnamic acid [40,41]; liquiritigenin derivatives [42]; carbamates [43,44,45]; plant-derived compounds [46]; N-aryl compounds [47,48]; oximes, N-hydroxy-iminoacetamides, 4-aminoquinolines [49]; flavonoids [50,51], cardiovascular drugs, and benzodiazepines [52]; pyrizadinones [53]; p-aminobenzoic acid derivatives [54]; benzimidazoles [55,56]; 1,2,3-triazoles [57]; piperidinehydrazide-hydrazones [58]; N-benzylpyrrolidines [59]; biphenyls, bibenzyls and benzylbenzenes [60,61,62]; arylthiourea derivatives [36]; physostigmine derivatives [63,64]; phenyl pentenone derivatives [65]; and N-benzylpiperidine, indanone, quinazolinimine, and bis-pyridinium derivatives [34].

As concluded from the analysis of published models (even those involving small sets of structurally related compounds), the ability of compounds to inhibit AChE is not a linear function of their easily explainable physicochemical properties (e.g., lipophilicity, molecular size, or polarity).

Relatively few reported models of AChE inhibition are from a 2D QSAR group, based on thermodynamic, topological, graph-theoretical, steric, and electronic descriptors, for example: (i) N composition, maximal projection area, volume, log ***D*,** and polar water-accessible surface area [66]; (ii) ***Mor06v***, ***G1u***, ***GATS2e***, and ***R5e*** [38]; (iii) ***WIENER***, ***ALOGP98***, ***CHI-V-3_C***, and ***PHI*** [48]; (iv) volume, HOMO-LUMO energy gap, LUMO energy, rotatable bond count, molar refractivity, intracycle oxygen/nitrogen, and non-aromatic ring [61]; (v) energy bandgap (***ΔE)***, total volume of cavity (***TVC***), dipole moment (***DM***), topological polar surface area (***TPSA***), molecular weight (***MW***), partition coefficient (log ***P***), hydrogen bond acceptors (***HBAs***), and chemical potential (***μ***) [62]; (vi) ***nOH***, ***nR10***, and ***0χν*** [49]; (vii) ***DM***, ***PSA***, solvent-accessible volume, log ***S***, and molecule length [67]; (viii) Balaban Index, total energy, dipole moment, and electrophilicity index [42]; (ix) the energy of the second highest occupied molecular orbital, dipole moment, %carbon, and rotatable bond count [44]; (x) Connolly Accessible Area, ***E***_LUMO_, and ***Hydrogen%*** [45]; (xi) ***HVcpx***, ***EA2***, ***HATS5u***, and ***qpmax*** [40]; (xii) N composition, maximal projection area, volume, log ***D***, and water-accessible polar surface area [66].

Many published AChE ***IC***_50_ or ***K***_i_ models involve a 3D QSAR approach [60,66,68,69], particularly ligand-based methods such as CoMFA (Comparative Molecular Field Analysis) [59] or CoMSIA (Comparative Molecular Similarity Indices Analysis) [67,70]. Attempts have also been made to study AChE inhibition using the 4D QSAR methodology [50]. Some models employ molecular fingerprints that represent the structural and chemical properties of molecules as fixed-length bit strings (binary vectors) or numerical vectors [71,72]. Molecular fingerprints are particularly useful for analyzing large sets of compounds [73,74,75].

In this study, we targeted 86 veterinary antiparasitic drugs from different chemical families. We investigated compounds that have been previously studied in the context of their membrane permeability, environmental distribution, absorption from the gastrointestinal tract, and blood–brain barrier permeability [76]. In this study, we focused on the ability of these drugs to cross the human placenta and interact with human AChE.

## 2. Results

### 2.1. Antiparasitic Drugs’ Ability to Cross the Placenta

#### 2.1.1. General Considerations

In vivo studies of placental permeability in pregnant women cannot be conducted for ethical reasons. Unlike other biological barriers (e.g., skin and blood–brain barrier), results from animal models are not easily extrapolated to humans due to significant interspecies variation [77]. One of the most valuable methods for assessing placental transfer is the perfusion of a single human cotyledon. This in vitro methodology and its subsequent modifications enable the study of compounds’ ability to cross the placenta using human tissues without posing health risks to mothers or children. Placental transfer measured using perfusion methods is usually expressed as the Clearance Index (***CI***) or Transfer Index (***TI***), with antipyrine used as a reference compound to account for inter-placental variations (antipyrine is a small, lipophilic molecule that does not bind to proteins and crosses the placenta very easily by passive diffusion). Earlier QSAR studies indicated that the ***CI*** or ***TI*** are positively correlated with lipophilicity (log ***P***), heat of formation, number of 5-membered rings, number of ethyl groups, greatest positive partial atomic charge, and halogen count in a molecule, and inversely correlated with ***TPSA*** and the count of H-bond acceptors. However, the MLR models developed to discover these relationships were based on relatively small sets of reference compounds [77,78,79]. A more general PLS model of the ***CI*** reported by Giaginis et al. [80] corroborated these results and showed that the ***CI*** is also inversely correlated with the tendency to form H-bonds, ionization, and dipole moment.

#### 2.1.2. Giaginis Reference Set vs. Studied Compounds

**Figure 1:**
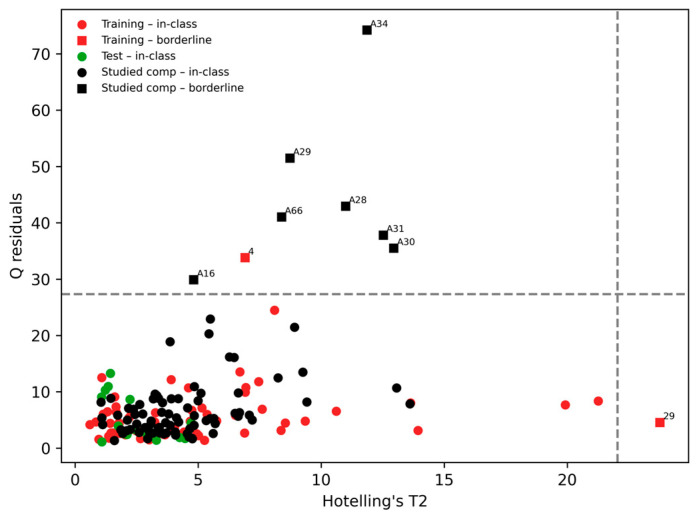
Q residuals vs. Hotelling’s T^2^ plot for studied compounds and Giaginis reference set.

The descriptor space of the studied compounds was compared with that of the reference compounds using Principal Component Analysis (PCA). Principal components were derived from a set of descriptors commonly associated with compounds’ ability to cross biological barriers (Supplementary Materials). Hotelling’s T^2^ (the score distance, expressed as the squared Mahalanobis distance from the origin of the score subspace) and Q (the orthogonal distance, expressed as the sum of squared residuals) [81,82] were computed for the reference compounds (training set), and plotted in the Q vs. T^2^ coordinate system, with the test set and the studied compounds superimposed on this plot (Figure 1). It was established that seven antiparasitic drugs (including some salicylamides) and one compound from the reference set have Q residuals exceeding the threshold; these compounds are atypical, so the ***CI*** predictions for them may be less accurate than those for the others. One compound from the reference group has an elevated T^2^ value. No studied drugs exceeded both thresholds, indicating sufficient similarity between the physicochemical properties of the reference set and those of the studied compounds to justify using the Giaginis reference dataset in this study.

#### 2.1.3. MLR Models of Placental Permeability

**Figure 2:**
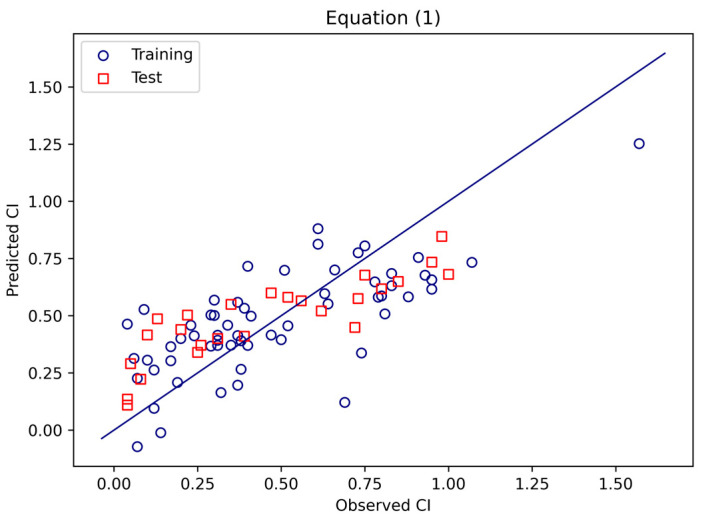
CI—predicted vs. experimental values, Equation (1).

**Figure 3:**
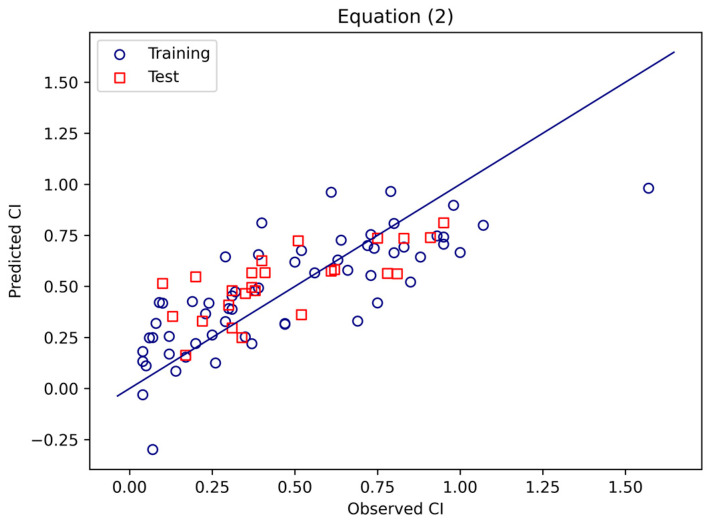
CI—predicted vs. experimental values, Equation (2).

**Figure 4:**
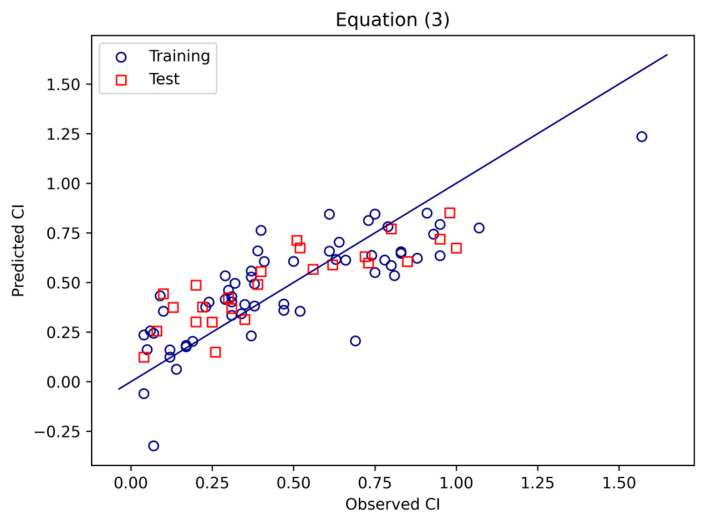
CI—predicted vs. experimental values, Equation (3).

**Figure 5:**
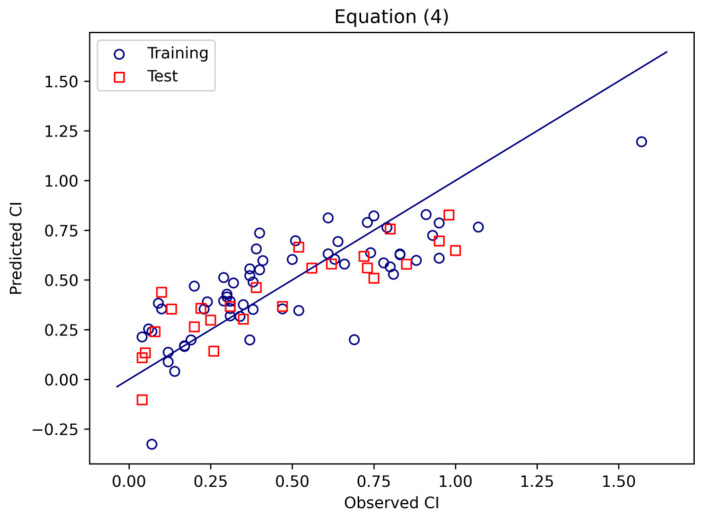
CI—predicted vs. experimental values, Equation (4).

Initially, the placental clearance index (***CI***) of 86 antiparasitic drugs was predicted using Equation (1) (Figure 2) and Equation (2) (Figure 3), and their features were combined to furnish Equation (3) based on the following descriptors: ***caco2***, ***TPSA***_Mord_, ***nHet***, and ***ATSC7i*** (Figure 4). After removing the least influential variable ***nHet*** (accounting for only approximately 2% of the total variability), Equation (4) was obtained (Figure 5).

***CI*** = 1.216 (±0.171) − 0.00246 (±0.00037) ***TPSA***_Mord_ + 0.00291 (±0.00116) ***ATSC7i*** − 0.404 (±0.157) ***GATS3m*** (1)

(n = 60, R^2^ = 0.531, R^2^_adj._ = 0.507, F = 21.2, *p* < 0.01, RMSE_pred_ = 0.190, Q^2^_LOO_ = 0.384)

***CI*** = 2.505 (±0.315) + 0.0489 (±0.0301) ***nHD*** − 0.00556 (±0.00204) ***TPSA***_3.0_ + 0.0450 (±0.0205) ***nHet*** + 0.372 (±0.069) ***caco2*** (2)

(n = 60, R^2^ = 0.623, R^2^_adj._ = 0.600, F = 22.7, *p* < 0.01, RMSE_pred_ = 0.178, Q^2^_LOO_ = 0.491)

***CI*** = 2.173 (±0.288) + 0.00377 (±0.00923) ***nHet*** + 0.291 (±0.062) ***caco2*** + 0.00346 (±0.00100) ***ATSC7i*** − 0.00138 (±0.00046) ***TPSA***_Mord_ (3)

(n = 60, R^2^ = 0.659, R^2^_adj._ = 0.634, F = 26.5, *p* < 0.01, RMSE_pred_ = 0.171, Q^2^_LOO_ = 0.436)

***CI*** = 2.173 (±0.295) + 0.295 (±0.062) ***caco2*** + 0.00346 (±0.00101) ***ATSC7i*** − 0.00118 (±0.00042) ***TPSA***_Mord_ (4)

(n = 60, R^2^ = 0.626, R^2^_adj._ = 0.606, F = 31.2, *p* < 0.01, RMSE_pred_ = 0.166, Q^2^_LOO_ = 0.461)

**Figure 6:**
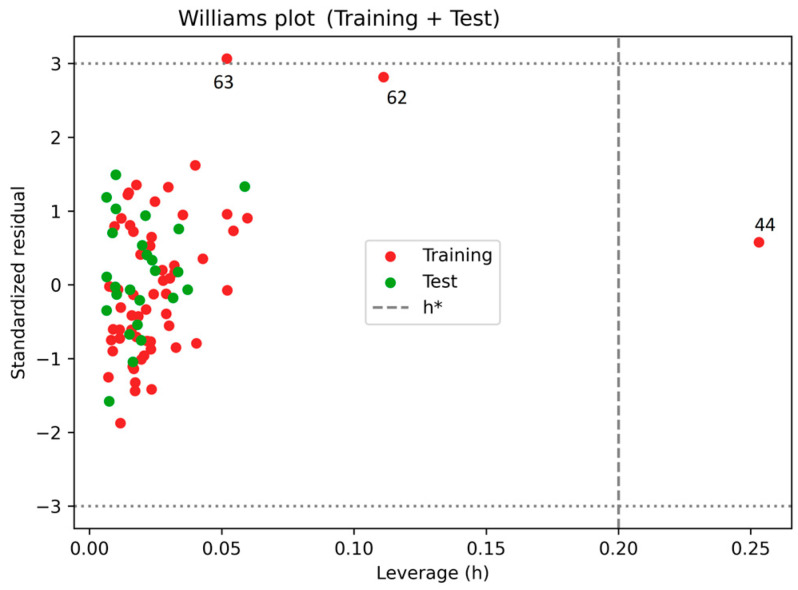
Williams plot for Equation (4).

**Figure 7:**
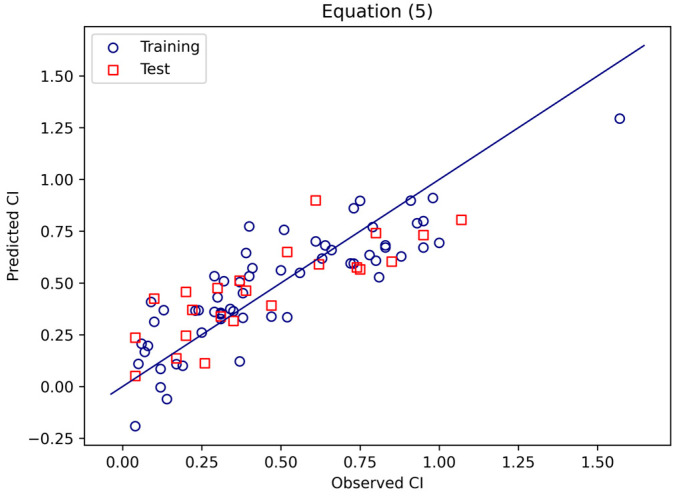
CI—predicted vs. experimental values, Equation (5).

Equation (4) was examined using the standard residual (SR) vs. leverage (h) plot (a so-called Williams plot) [83,84] (Figure 6; Supplementary Materials). It was revealed that three compounds in the training set (44, 62, and 63) differ from the others. Compounds 62 and 63 have the standardized residuals slightly below and above the SR threshold (|SR|= 3), respectively, and compound 44 has a leverage value higher than the h* threshold, but its standard residual is within the limits, so it has a significant influence on the model. No compounds in the reference set are both response outliers and high-leverage compounds [83]; nevertheless, the ***CI*** values for compounds 62 (ouabain) and 63 (riboflavin) were considered questionable (riboflavin was identified as an outlier in other placental ***CI*** models based on the same dataset, Ref. [80]). Both values were initially reported by the same authors [85,86], suggesting that these measurements might not be fully comparable, in methodological terms, to the remaining ***CI*** values in the dataset. Finally, a decision was made to remove compounds 62 and 63 from further studies, and the improved version of Equation (5) was obtained (Figure 7).

*CI* = 2.154 (±0.286) + 0.273 (±0.061) *caco2* + 0.00382 (±0.00088) *ATSC7i* − 0.00195 (±0.00040) *TPSA*_Mord_ (5)

(n = 60, R^2^ = 0.734, R^2^_adj._ = 0.720, F = 51.5, *p* < 0.01, RMSE_pred_ = 0.170, Q^2^_LOO_ = 0.647

**Figure 8:**
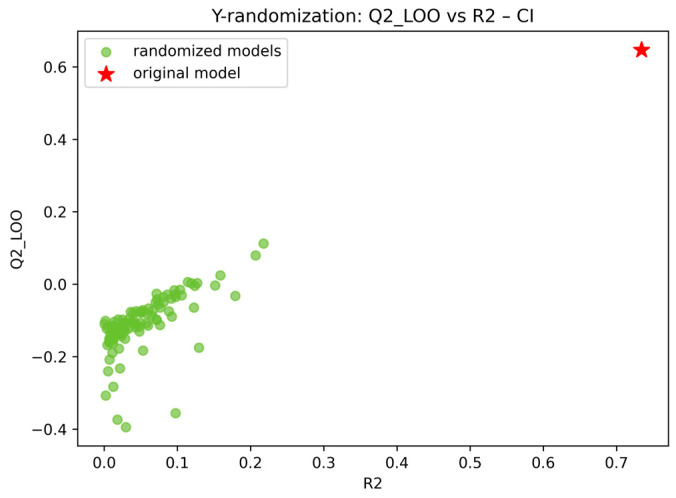
Y-randomization test, Equation (5).

The robustness of Equation (5) was tested using the Y-randomization approach, comparing the performance of the original model (R^2^) with that of models built on permuted responses, using the original descriptor pool and the original model building procedure [87] (Figure 8; Supplementary Materials). The possibility of chance correlation between ***CI*** and the descriptors used in Equation (5) was ruled out because the performance of the newly generated models (Q^2^_LOO_, R^2^) was markedly worse than that of the original model.

It was established that in the MLR equation (5), the ***CI*** is positively correlated with ***caco2***, the calculated apparent permeability across a monolayer of Caco-2 cells (log ***P***_app_), defined as follows [88]:

Papp= dQdtC0·A

where d***Q/***d***t*** is the rate of permeation across the cells, ***C***_0_ is the donor concentration at time zero, and ***A*** is the area of the cell monolayer.

According to Equation (5), the ***CI*** is inversely correlated with the (topological) polar surface area (***TPSA)***, a descriptor known to limit the ability of compounds to cross biological barriers, such as (i) the blood–brain barrier [89]; (ii) the intestinal barrier [90]; and (iii) the placenta [77]. The ***CI*** is also positively correlated with ***ATSC7i***—the centered Broto-Moreau autocorrelation—lag 7/weighted by the first ionization potential, which has already been used in some QSAR models, for example, to predict aromatase inhibition by indole–azole analogs [91]. The ***CI*** values predicted for 86 antiparasitic drugs are listed in Table 1.

### 2.2. MLR Models of Drugs’ Ability to Inhibit AChE

**Figure 9:**
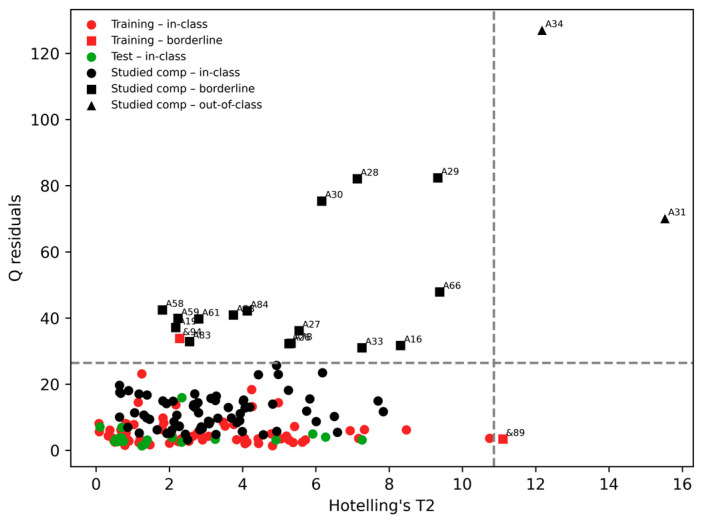
Q residuals vs. Hotelling’s T^2^ plot for studied compounds and p**K**_i_ reference set.

The ability of compounds to inhibit enzymes may be expressed by their 50% inhibitory concentration (***IC***_50_) or inhibition constant (***K***_i_). The inhibition constant is an equilibrium dissociation constant that reflects the binding affinity of a ligand for its binding partner, thereby reducing the binding partner’s activity. ***K***_i_ represents the concentration at which the inhibitor ligand occupies 50% of the receptor sites in the absence of a competing ligand [92]. In this study, the logarithmic inhibition constants (p***K***_i_) of 86 antiparasitic drugs were predicted using linear models based on straightforward descriptors calculated for a set of reference compounds. The descriptor space of the reference compounds was compared with that of the studied drugs in the Q residuals–Hotelling T^2^ coordinate system (Figure 9; Supplementary Materials). It was observed that 16 of 86 studied compounds (in particular, salicylanilides) can be classified as “borderline” because their Q residuals are elevated, indicating atypical properties. Therefore, the p***K***_i_ values predicted for these compounds may be less accurate than those for the others. Two studied drugs (A34—bromofenofos and A31—nitroxinil) are out-of-class, i.e., both their Q and T2 values exceed the thresholds. Therefore, predictions of their p***K***_i_ values using the models developed in this study may be unreliable.

**Figure 10:**
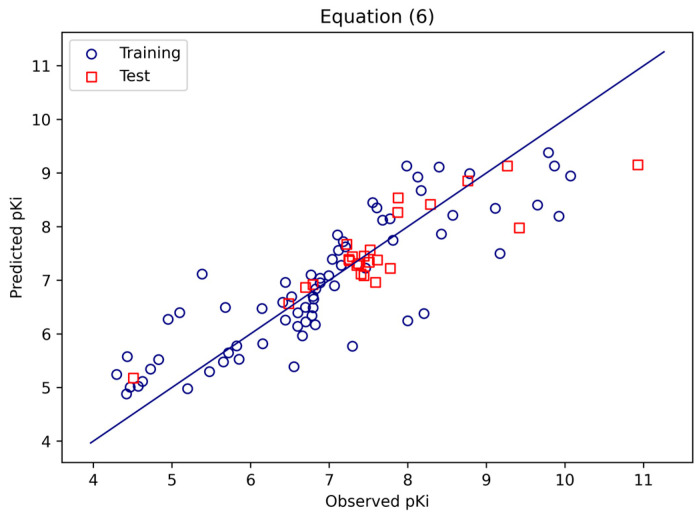
p**K**_i_—predicted vs. experimental values, Equation (6).

**Figure 11:**
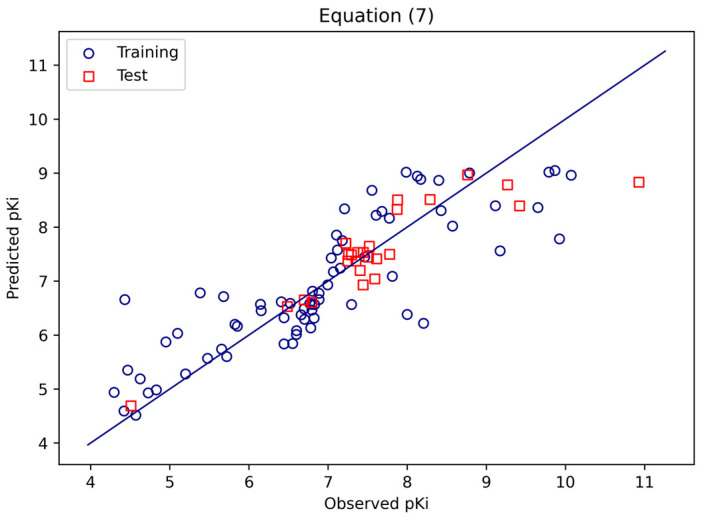
p**K**_i_—predicted vs. experimental values, Equation (7).

**Figure 12:**
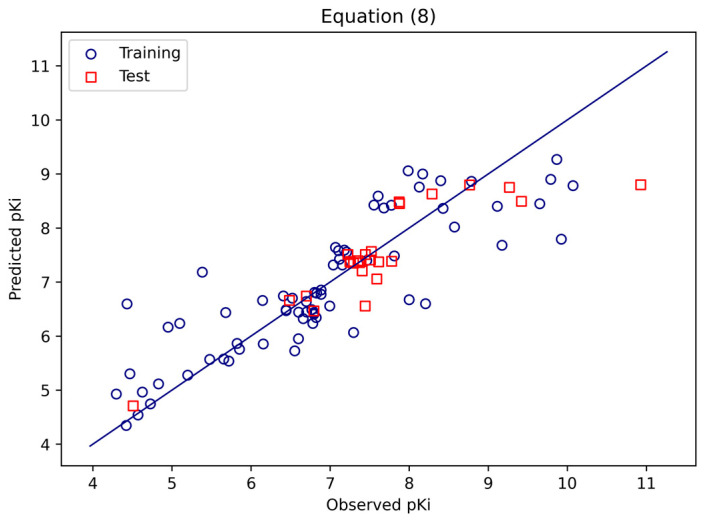
p**K**_i_—predicted vs. experimental values, Equation (8).

**Figure 13:**
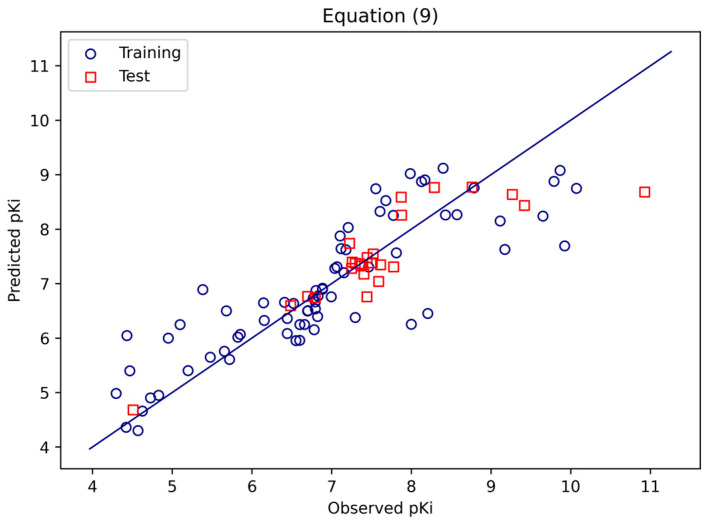
p**K**_i_—predicted vs. experimental values, Equation (9).

At this stage of our study, four MLR models for p***K***_i_ were selected from a set of 378 candidates generated, as described in Section 4, based on their R^2^_train, R^2^_test, and RMSE_test values, with the primary goal of balancing internal and external validation results (Equations (6)–(9); Figure 10, Figure 11, Figure 12 and Figure 13).

p***K***_i_ = 15.26 (±1.44) + 0.354 (±0.067) ***nRing*** + 2.78 (±0.57) ***Fsp3*** + 2.20 (±0.31) ***caco2*** − 0.0180 (±0.0063) ***Fu*** + 0.835 (±0.299) ***logVDss*** (6)

(n = 70, R^2^ = 0.721, R^2^_adj._ = 0.699, F = 33.1, *p* < 0.01, RMSE_pred_ = 0.553, Q^2^_LOO_ = 0.672)

p***K***_i_ = 10.10 (±2.26) + 0.394 (±0.067) ***nRing*** + 3.10 (±0.53) ***Fsp3*** + 1.53 (±0.31) ***caco2*** − 0.00969 (±0.00325) ***TPSA***_3.0_ + 0.00688 (±0.00297) ***bp*** (7)

(n = 70, R^2^ = 0.710, R^2^_adj._ = 0.688, F = 31.14, *p* < 0.01, RMSE_pred_ = 0.550, Q^2^_LOO_ = 0.657)

p***K***_i_ = 13.15 (±1.73) + 0.475 (±0.051) ***nRing*** + 3.11 (±0.52) ***Fsp3*** + 1.71 (±0.39) ***caco2*** − 0.00950 (±0.00316) ***TPSA***_3.0_ − 0.0190 (±0.0062) ***Fu*** (8)

(n = 70, R^2^ = 0.726, R^2^_adj._ = 0.705, F = 33.93, *p* < 0.01, RMSE_pred_ = 0.570, Q^2^_LOO_ = 0.680)

p***K***_i_ = 12.11 (±1.79) + 0.425 (±0.055) ***nRing*** + 2.58 (±0.54) ***Fsp3*** + 1.62 (±0.40) ***caco2*** − 0.00770 (±0.00330) ***TPSA***_3.0_ + 0.177 (±0.062) ***logP*** (9)

(n = 70, R^2^ = 0.722, R^2^_adj._ = 0.700, F = 33.24, *p* < 0.01, RMSE_pred_ = 0.593, Q^2^_LOO_ = 0.673)

**Figure 14:**
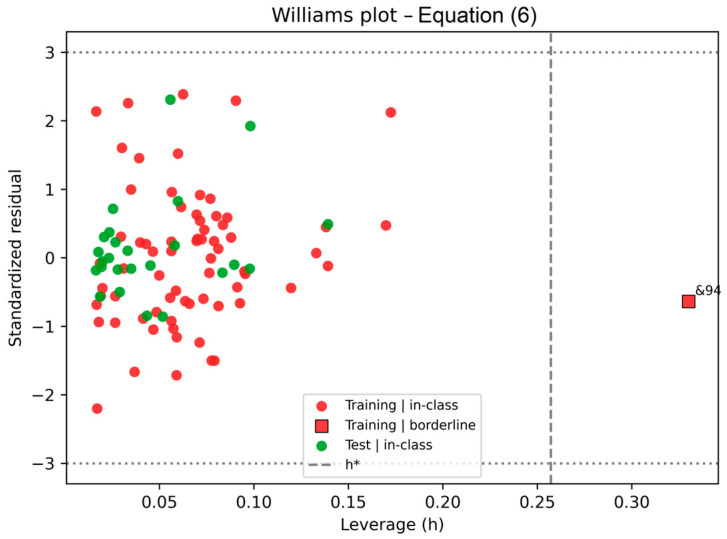
Williams plot for Equation (6).

**Figure 15:**
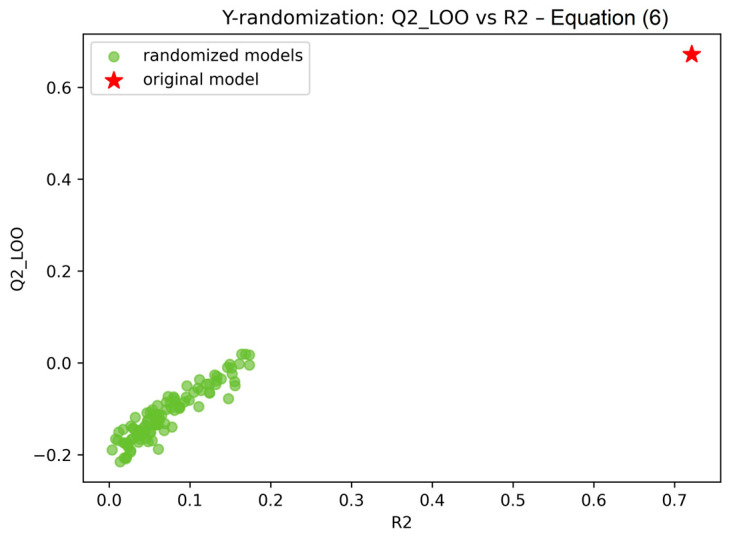
Y-randomization test, Equation (6).

**Figure 16:**
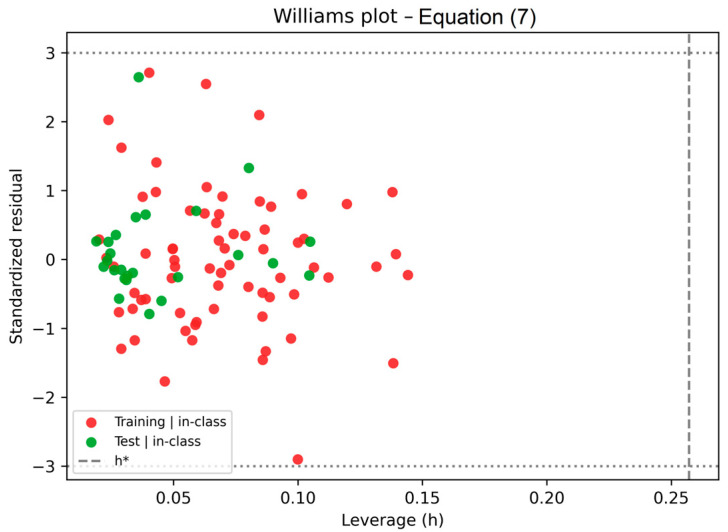
Williams plot for Equation (7).

**Figure 17:**
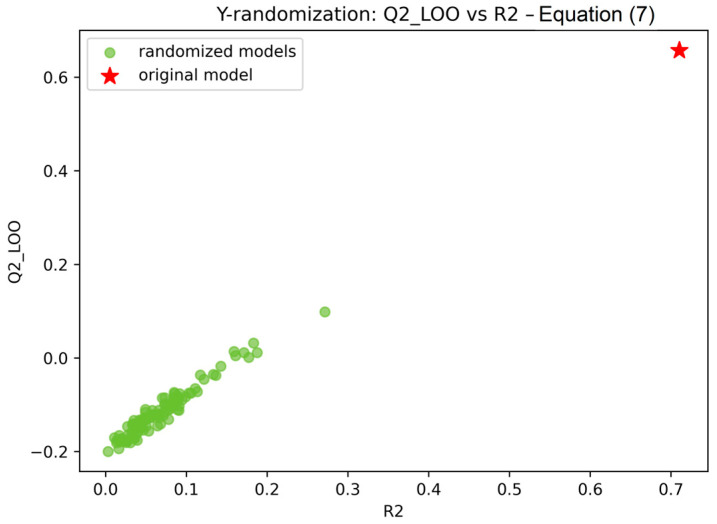
Y-randomization test, Equation (7).

**Figure 18:**
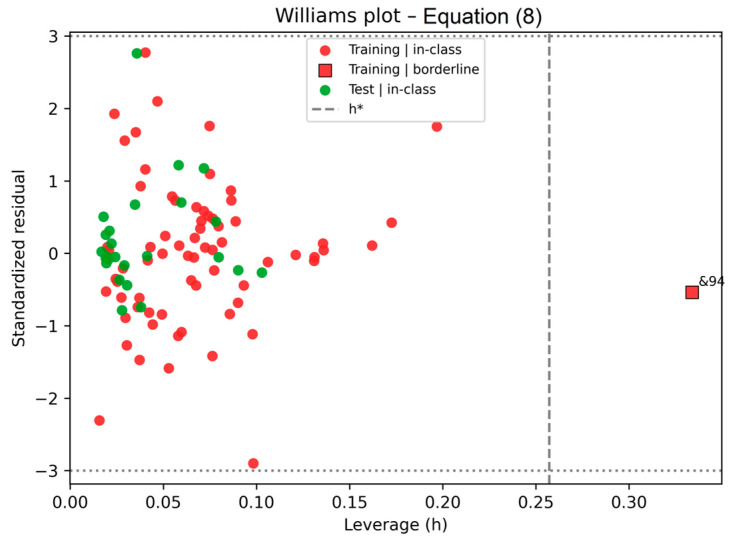
Williams plot for Equation (8).

**Figure 19:**
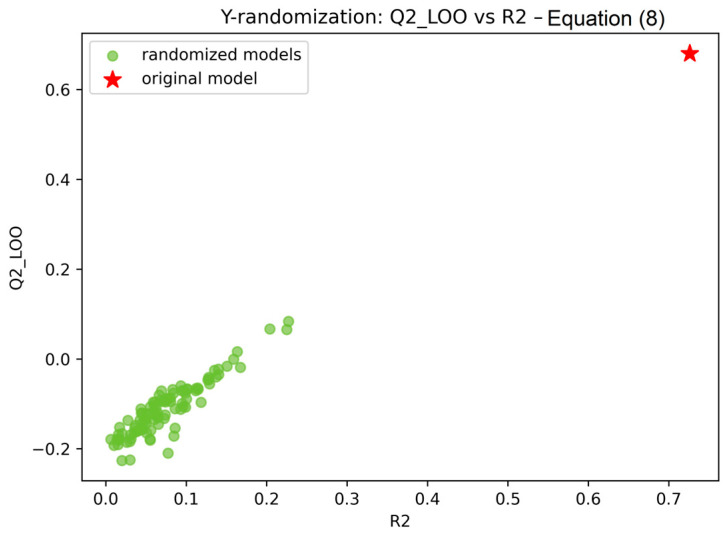
Y-randomization test, Equation (8).

**Figure 20:**
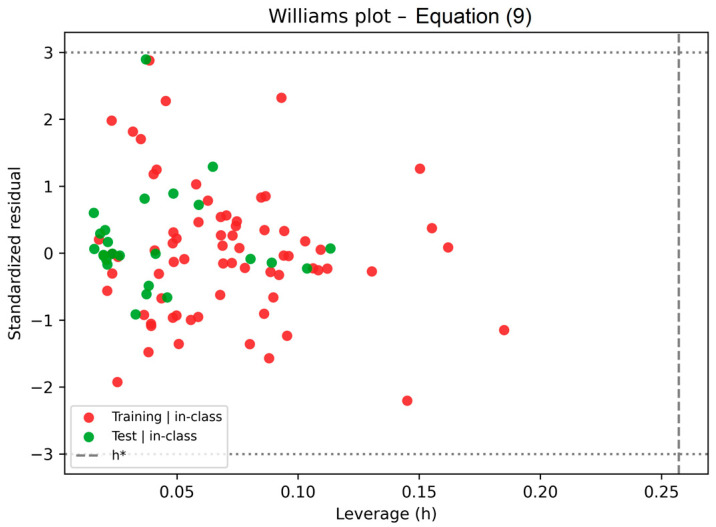
Williams plot for Equation (9).

**Figure 21:**
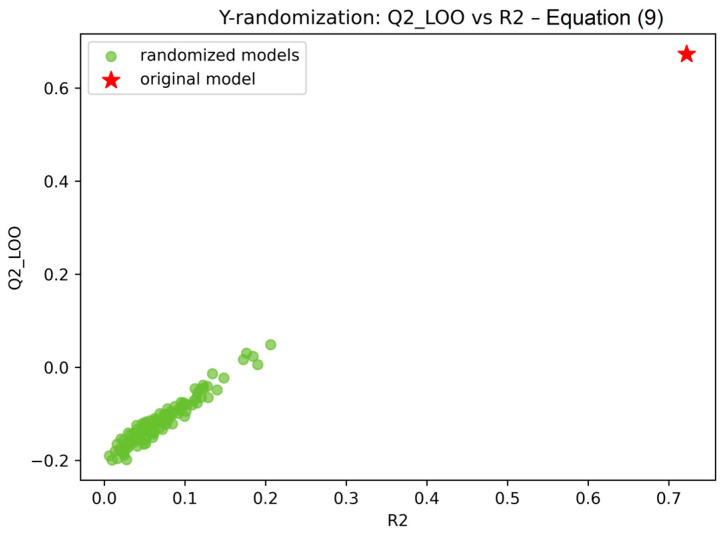
Y-randomization test, Equation (9).

In all models considered in the final stages of this study, p***K***_i_ is positively correlated with ***caco2***, ***nRing***, and ***Fsp3*** (these descriptors account for almost 64% of the total variability); other (less common and less significant) variables associated with higher p***K***_i_ are ***logVDss***, ***logP***, and ***bp***. Variables inversely correlated with p***K***_i_ are ***TPSA*** and ***Fu***. In other words, molecules are expected to be stronger AChE inhibitors if they are more flexible (higher ***Fsp3***), have a higher ring count (***nRing***), or exhibit higher Caco-2 permeability (***caco2***). More polar molecules (***TPSA***) or those with a higher ability to bind to plasma proteins (lower ***Fu***) are, according to Equations (6)–(9) (Figure 14, Figure 15, Figure 16, Figure 17, Figure 18, Figure 19, Figure 20 and Figure 21), expected to have a lower affinity for AChE. Equations (6)–(9) were evaluated using Williams plots (Figure 14, Figure 16, Figure 18 and Figure 20; Supplementary Materials), and their robustness was tested by y-randomization (Figure 15, Figure 17, Figure 19 and Figure 21; Supplementary Materials). It was established that no compounds in the reference set are outliers (although two compounds, &65 and &74, have relatively high standardized residuals in Equation (9), and compound &44 is close to the lower SR limit in Equations (7) and (8)). One compound (&94) is highly influential in some models (Equations (6) and (8)).

**Figure 22:**
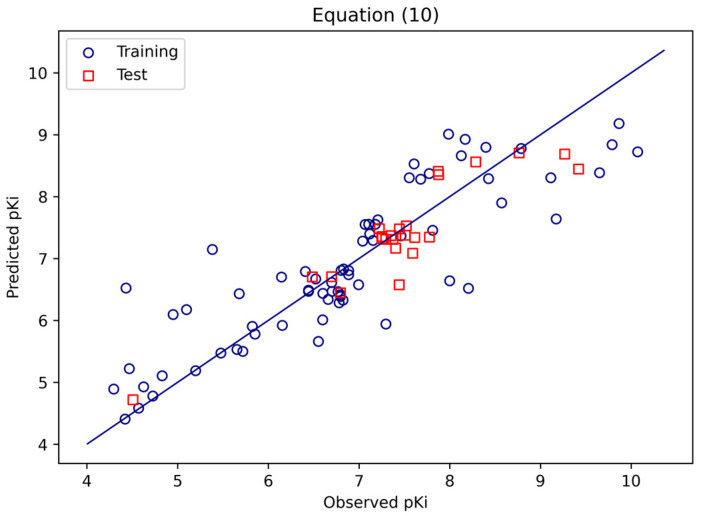
p***K***_i_—predicted vs. experimental values, Equation (10).

**Figure 23:**
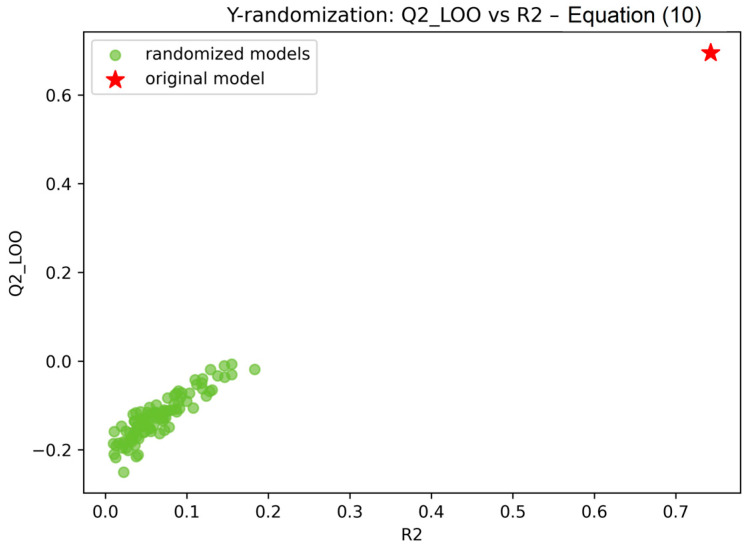
Y-randomization test, Equation (10).

Compounds &65, &74, and &44, technically speaking, fit the AD, but in search of a more stable model, they were removed; the independent variables from Equations (6)–(9) were combined and processed using stepwise regression (forward mode) to yield Equation (10) (Figure 22 and Figure 23), which encompasses the same descriptors as Equation (8), but with improved statistical parameters.

p***K***_i_ = 13.71 (±1.64) + 0.548 (±0.048) ***nRing*** + 3.042 (±0.490) ***Fsp3*** + 1.818 (±0.372) ***caco2*** − 0.0180 (±0.0059) ***Fu*** − 0.00872 (±0.00299) ***TPSA*** (10)

(n = 69, R^2^ = 0.743, R^2^_adj._ = 0.722, Q^2^_LOO_ = 0.700, RMSE_pred_ = 0.383, F = 36.35, *p* < 0.001)

### 2.3. Molecular Docking of Antiparasitic Drugs

Molecular docking is a common approach for rapid screening and comparison of ligand affinity for biological targets, including acetylcholinesterase. The AChE crystal structure most frequently used in docking studies is 4EY7 (a complex of human AChE with a reference AChE inhibitor, donepezil) [93]. Based on this structure, 27 possible binding pockets were identified in chain A of the enzyme dimer; one of which, involved in donepezil binding, was used in the molecular docking calculations performed in this study. Thus, drug–protein affinities were calculated, and protein–ligand interactions were visualized (Supplementary Materials).

## 3. Discussion

### 3.1. Placenta Clearance Index

**Figure 24:**
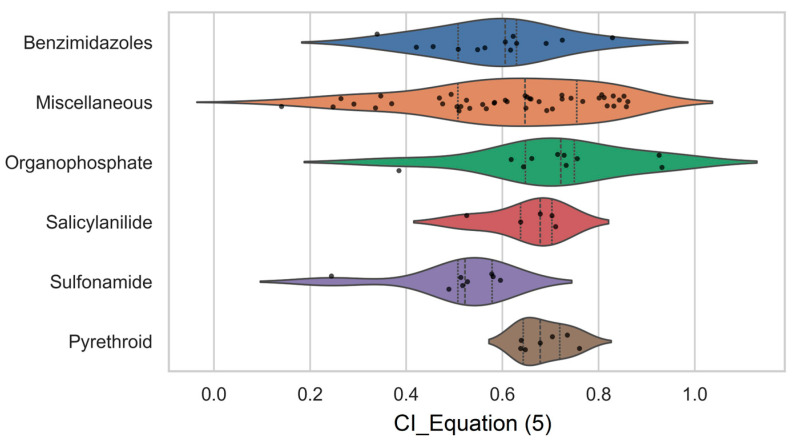
Comparison of CI values for different chemical families of antiparasitic drugs.

It was established (Table 1 and Figure 24) that several drugs from the studied group (phoxim, diazinone, pyrantel, morantel, levamisol, diethylcarbamazine, amitraz, carbaryl, thiabendazole, epsiprantel, pyriproxifen, propoxur, and praziquantel) have a high placental ***CI*** relative to antipyrine (***CI*** ≥ 0.8). According to the predicted ***CI*** values, the rate of passage across the placenta should be moderately restricted for ten drugs: bromofenofos, halofuginone, lufenuron, netobimin, dinitolmide, niclofolan, pentamidine, diminazene, clorsulone, and amicarbalide (predicted ***CI*** values < 0.40). ***CI*** values differed to some extent across chemical families; they were, on average, lower for sulfonamides than for drugs from other chemical families. However, because the drugs under study are structurally diverse within the chemical families, no general conclusions can be drawn in this respect.

Relatively high ***CI*** values predicted for pyrethroids (between 0.60 and 0.80) are at odds with their low fetus-to-mother concentration ratio (***FM***) values, as evaluated in a previous study [94]. However, the ***CI*** and ***FM*** differ in methodology and meaning. The ***CI*** provides useful insights into the mechanistic and kinetic aspects of transplacental passage, whereas the ***FM*** represents the steady-state distribution of a compound between the fetal and maternal circulations, accounting for placental or fetal metabolism and non-placental clearance routes. Pyrethroids are rapidly metabolized and are unlikely to bioaccumulate [29], which affects their equilibrium concentrations in the maternal and fetal compartments.

### 3.2. Affinity for AChE

The ability of the studied drugs to interact with human AChE was evaluated using inhibition constants (***K***_i_) predicted by MLR models (in particular, Equation (10)), which incorporated easily interpretable physicochemical and membrane permeability descriptors, selected from a larger set (“best subset” approach).

**Figure 25:**
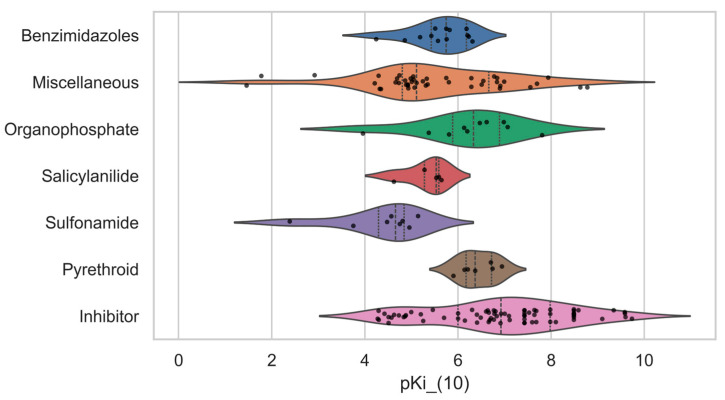
Comparison of p**K**_i_ values for different chemical families of antiparasitic drugs.

The predicted p***K***i values for 86 antiparasitic drugs varied across chemical families; on average, they were slightly higher for organophosphates and pyrethroids than for other groups (Figure 25). Almost all values, except for six (bromofenofos, sulfadiazine, pentamidine, clorsulone, diminazene, and amicarbalide), fell within the range observed for AChE inhibitors in the CHEMBL database. Based on the predicted p***K***_i_ values, the potential of antiparasitic drugs to inhibit human AChE warrants further attention.

**Figure 26:**
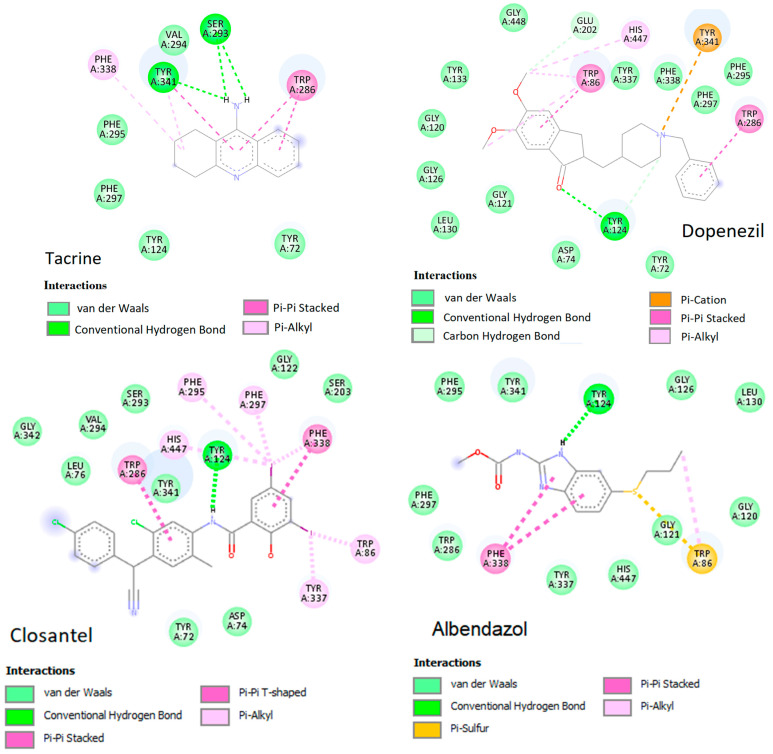

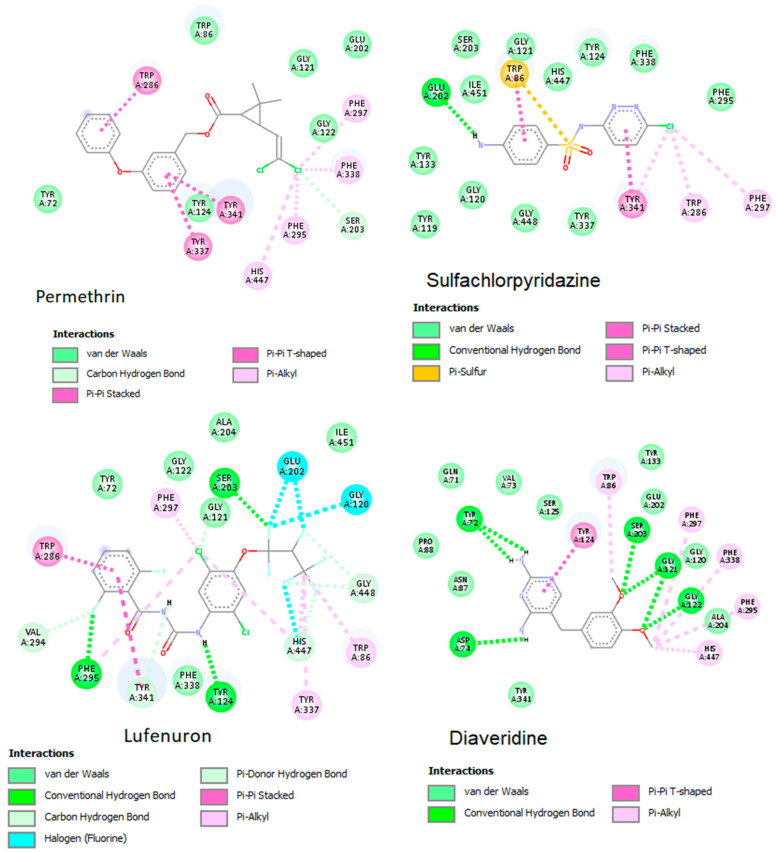
Predicted AChE–ligand interactions for the selected drugs and known AChE inhibitors.

**Figure 27:**
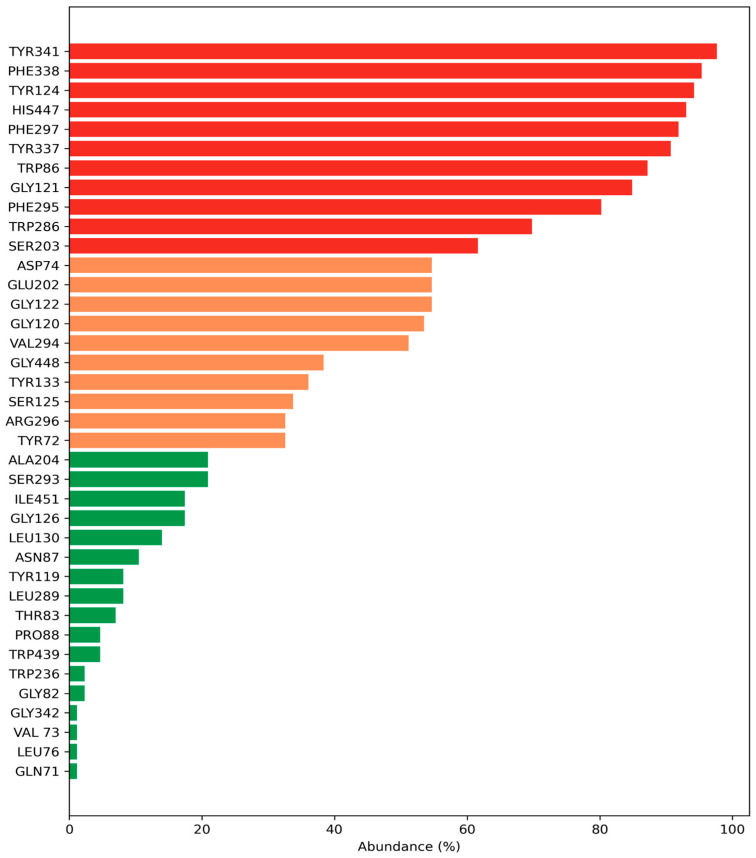
An abundance of interactions of antiparasitic drugs with the particular amino acids.

The mechanism of antiparasitic drug binding to AChE was investigated theoretically by non-covalent molecular docking. A large number of protein–ligand interactions were predicted, including conventional H-bonds, C-H bonds, halogen (fluorine, in particular), π-π and π-alkyl interactions, π-donor hydrogen bonds, π-sulfur or π-σ interactions, and van der Waals bonds (Figure 26 and Supplementary Materials). AChE encompasses multiple binding sites: a catalytic site/triad [95,96] (Ser203, His447 and Glu334); an anionic subsite (Tyr86, Tyr133, Tyr 337, Phe338), an acyl-binding pocket (Phe298, Phe297, Trp236), an oxyanion hole (Gly121, Gly122, Ala204) and a peripheral anionic site (Tyr72, Asp74, Tyr124, Trp 286, Tyr 341) [97,98]. The majority of drugs analyzed in our study are expected to interact with at least one of the amino acids engaged in the AChE catalytic triad (abundance of interferences with the particular amino acid residues in the studied group of antiparasitic drugs is given in %): His447—93% and Ser203—62% (Figure 18; Supplementary Materials). Other amino acids engaged in the enzyme–ligand interactions with almost all the studied drugs were tryptophan (Trp86—87%, Trp236—70%, Trp286—69%), tyrosine (Tyr124—95%, Tyr227, Tyr341—97%, Tyr72—33%), phenylalanine (Phe295—80%; Phe297—92%, and Phe338—95%), glycine (Gly 121—85%, Gly122—55%), and, occasionally, alanine (Ala204—20%) (Figure 27). Similar interactions were predicted for the AChE inhibitors currently (or formerly) used to treat dementia in humans (tacrine, donepezil, and physostigmine). In addition to protein–ligand interactions that contribute to complex formation, unfavorable acceptor–acceptor or donor–donor interactions are also present in some cases. These interactions may reduce the stability of enzyme-drug complexes over time for cypermethrin and amitraz (Tyr124); oxfendazole, netobimin, luxabendazole, carbaryl, and sulfadimethoxine (Ser203); sulfadiazine and sulfamethoxazole (Gly120); and diamfenetide (Asn87).

## 4. Materials and Methods

### 4.1. Reference Compounds

Placental clearance index (***CI***) values (relative to antipyrine) for 85 compounds (1 to 85) measured using the ex vivo human placental perfusion method were compiled by Giaginis [80]. Outliers were identified using the Williams plot (standardized residuals vs. leverage) generated for an initial model (Equation (4)). Two compounds, ouabain (Compound 62) and riboflavin (Compound 63), had standardized residuals of approximately 3 and were not high-leverage points, indicating inconsistent ***CI*** values relative to the rest of the data. These two compounds were therefore excluded, and the final model (Equation (5)) was developed for 83 compounds.

The experimental human AChE inhibition constants (***K***_i_) were obtained from the CHEMBL database [99,100] (target enzyme symbol—CHEMBL220). The initial dataset comprised 654 records from 127 sources (scientific papers). Because it contained values measured using different protocols, with poor reproducibility, multiple repetitions, and inconsistencies, it was of no value in its raw form. The smallest subsets of ***K***_i_ values (fewer than 10 records from a single source) were removed, and the p***K***_i_ values from the remaining 17 papers were used as input for MLR analysis (“best subset” mode) in Python 3.13. MLR models with 3 to 5 independent variables were generated, and compound subsets from the same source that had the greatest negative impact on model performance were removed blockwise in 10 steps, followed by manual duplicate removal. Hence, 95 unique compounds (&1 to &95)with known ***K***_i_ values [101,102,103,104,105,106,107] remained (Supplementary Materials).

### 4.2. Calculated Molecular Descriptors and Membrane Permeability Data

**Figure 28:**
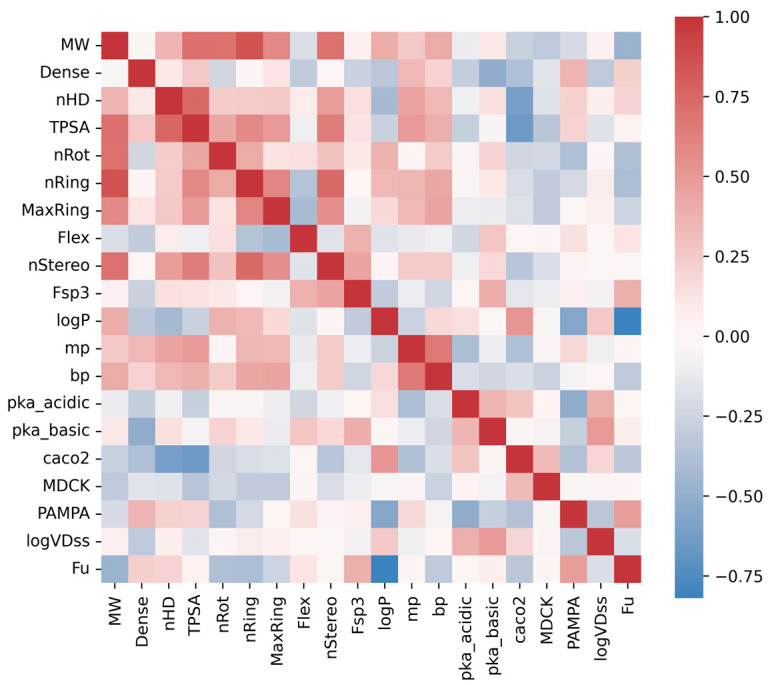
Correlations between the descriptors—***CI*** training set.

**Figure 29:**
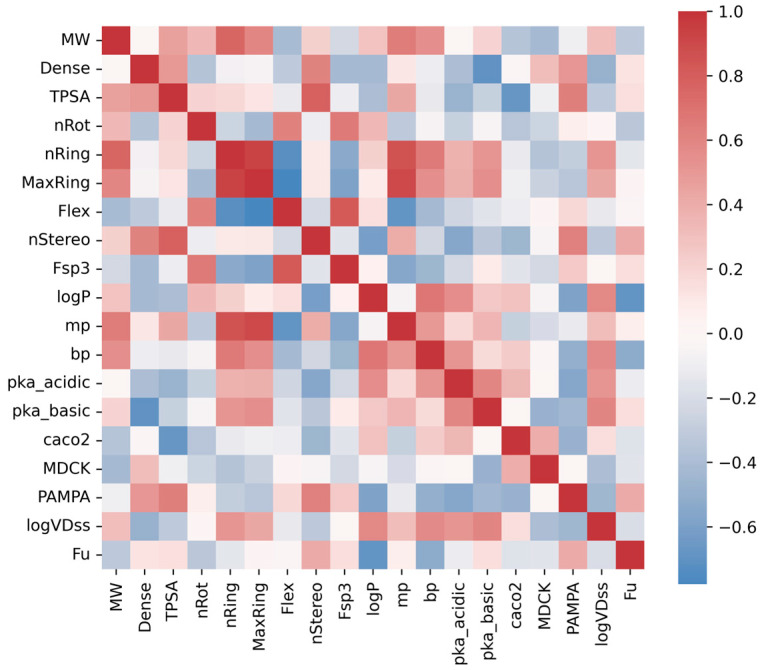
Correlation between the descriptors—p***K***_i_ training set.

The physicochemical and ADMET properties were calculated with ADMETLab3.0 [108] using canonical SMILES from PubChem or ChEMBL, which were used directly for descriptor calculation without further standardization or tautomer handling. The physicochemical descriptors considered relevant in this study were as follows: molecular weight (***MW***); van der Waals volume (***Vol***); density = ***MW***/***Vol*** (***Dense***); topological polar surface area (***TPSA***); the count of hydrogen bond acceptors (***nHA***); the count of hydrogen bond donors (***nHD***); the number of rotatable bonds (***nRot***); the number of rings (***nRing***); the number of atoms in the biggest ring (***MaxRing***); the number of non-carbon atoms (hydrogens included) (***nHet***); the number of rigid bonds (***nRig***); flexibility = ***nRot***/***nRig*** (***Flex***); the logarithm of aqueous solubility (**log*S***); the logarithm of the n-octanol/water partition coefficient (***logP***); the logarithm of the n-octanol/water distribution coefficients at pH = 7.4 (**log*D***); the number of sp^3^-hybridized carbons/total carbon count (***Fsp3***); melting point (***mp***); boiling point (***bp***); and dissociation constants (***pka_acidic***, ***pka_basic***). The ADMET properties considered in this study were as follows: ***caco2***; ***MDCK***; ***PAMPA***; the volume of distribution at a steady state (**log*VD*ss**); plasma protein binding, % (***PPB***); and the fraction unbound in plasma, % (***Fu***). A total of 1826 2D and 3D Mordred descriptors [109] were calculated using the OCHEM platform [110], with SMILES strings as input data and the Balloon algorithm for structure optimization [111]. The independent variables used to develop MLR models were not highly correlated (Figure 28 and Figure 29).

### 4.3. Multiple Linear Regression (MLR) Models

Multiple Linear Regression models reported in this study were developed in Python v. 3.13, using the reference compound sets described in Section 4.1. The reference sets for both endpoints (***CI*** and p***K***_i_) were split into training and test sets (with the test sets including compounds not used during model generation) using the Kennard–Stone algorithm [112]. The training and test sets comprised 60:25 compounds for the ***CI*** and 70:25 compounds for p***K***_i_, respectively.

MLR models of the ***CI*** were generated using descriptors from ADMETLab3.0 [108] and Mordred [109] (Supplementary Materials). Mordred descriptors were preselected using Statistica v. 13.3 from StatSoft, Kraków, via the stepwise Partial Least Squares (PLS) method based on Variable Importance in Projection (VIP) values. Variables with zero variation were discarded, and those with VIP ≥ 1 were considered relevant and used in the next step of the PLS analysis. The PLS procedure was repeated four times, with the number of PLS components selected by cross-validation each time, until the number of variables was reduced from 1826 to 28. The remaining 28 descriptors were processed using MLR (stepwise forward mode) to generate Equation (1), which included three key independent variables: ***ATSC7i***, ***GATS3m***, and ***TPSA***_Mord_ (Equation (1)). The ADMETLab 3.0 set of descriptors was reduced using the MLR method (stepwise forward mode) to four variables: ***caco2***, ***TPSA***_3.0_, ***nHet***, and ***nHD*** (Equation (2)), of which one (***nHD***) was the least influential.

The retained Mordred descriptors were combined with the ADMETlab 3.0 descriptors and processed using the MLR method in the “best subset” mode with the number of independent variables between 2 and 5 and the tolerance level set at 0.1 (it is assumed that two descriptors are collinear if the tolerance value between them, calculated as (1-R^2^), is <0.1 [113]) to furnish Equation (3) encompassing four independent variables: ***caco2***, ***TPSA***_Mord_, ***nHet***, and ***ATSC7i***. Equation (3) was refined by removing the least significant variable (***nHet***) and the outliers, as detected using an appropriate Williams plot (Figure 6).

MLR models of p***K***_i_ were selected from a set of 378 candidates generated during reference data curation (Section 4.1) based on R^2^_train, R^2^_test, and RMSE_test values, with the goal of identifying equations that could achieve satisfactory results in internal and external validation. The pre-selected models (no. 10—Equation (6); 14—Equation (7); 37—Equation (8); and 38—Equation (9)) underwent detailed validation.

### 4.4. Model Validation

Our validation approach follows the OECD principles for the regulatory use of (Q)SARs, by combining (i) a clearly defined endpoint; (ii) a fully specified algorithm; (iii) an explicitly characterized applicability domain (Q–Hotelling’s T^2^ analysis; Williams plots); (iv) comprehensive internal/external validation (including Kennard–Stone splitting, R^2^, Q^2^_LOO_, RMSE_pred_ and Y-randomization); and (v) a mechanistic interpretation of descriptor–endpoint relationships (where possible) [84].

### 4.5. Applicability Domain—Descriptor Spaces of Reference vs. Studied Compounds

**Figure 30:**
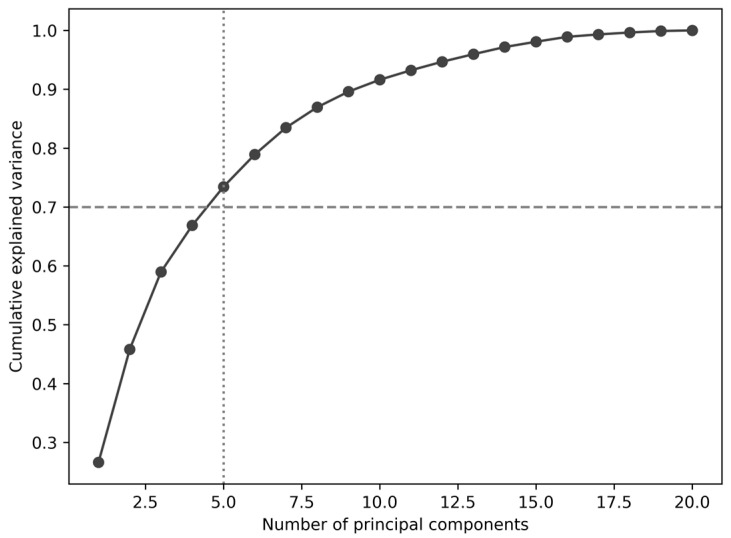
Cumulative variance vs. PC_no.—***CI*** training set.

**Figure 31:**
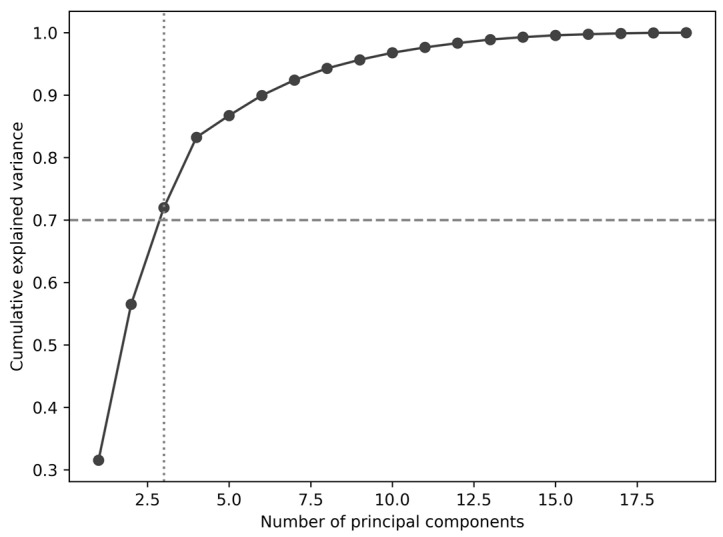
Cumulative variance vs. PC_no.—p***K***_i_ training set.

The Applicability Domain (AD) defines the chemical space within which model predictions can be considered valid [114]. There are several methods for determining AD [115,116,117]: (i) comparison of ranges in the descriptor space; (ii) geometrical methods; (iii) distance-based methods; (iv) probability density distribution; and (v) analysis of the range of the response variable. At first, we compared the descriptor spaces of the reference and studied compounds using Principal Component analysis [118], with the number of Principal Components set to account for at least 70% of the cumulative variance: 5 for the CI and 3 for pK_i_, respectively (Figure 30 and Figure 31). Hotelling’s T^2^ (the score distance, expressed as the squared Mahalanobis distance from the origin of the score subspace) and Q (the orthogonal distance, expressed as the sum of squared residuals) [81,82] were calculated for the training set, and the results were plotted in the Q vs. T^2^ coordinate system. The thresholds for Q residuals and Hotelling T^2^ were obtained from the distribution plots of both distance measures (alpha = 0.99), as shown in the Supplementary Materials. The test sets and the antiparasitics under study were superimposed on these plots. Compounds with Hotelling’s T^2^ or Q-residuals above the corresponding thresholds were flagged as “borderline”, whereas those exceeding both thresholds were classified as “out-of-class”. Predictions for borderline and out-of-class antiparasitic drugs are reported only for illustrative purposes.

### 4.6. Molecular Docking

Molecular docking was performed using MolModa 1.0.1 software from Durrant Labs [119] using the human AChE structure extracted as a PDB file from the RCSB Protein Data Bank (https://www.rcsb.org, accessed 2 January 2026): PDB ID—4EY7 (crystal structure of recombinant human acetylcholinesterase in complex with donepezil). The settings for drug–enzyme docking were as follows: sampling exhaustivity—8; box center coordinates: x = −16.3; y = −43.7; z = 28.6; box size: 22 × 10 × 17 Å.

The enzyme structure (chain A of a dimer, solvent, and other small molecules removed) was protonated using a suitable MolModa option.

The ligands were uploaded as SMILES strings retrieved from the PubChem database. Their SMILES strings were converted to 3D structures with maximum force-field optimization, protonated at pH 7.4 using the built-in Open Babel [120] functionality, and the 3D structures were optimized again with maximum force-field optimization.

The protein–ligand interactions of the AChE–ligand complexes were visualized using Discovery Studio 2024 software from Biovia (Supplementary Materials).

## 5. Conclusions

Humans can be exposed to veterinary antiparasitic drugs through several routes:

- The use of certain drugs against human endoparasites, ectoparasites, or protozoa.
- The misuse of preparations intended for pest control in domestic and farm animals; direct contact or residues in foods such as meat, eggs, and dairy products.
- Application as food additives (e.g., thiabendazole E233) to control fungal diseases in fruits (e.g., oranges, bananas) and vegetables.
- The reported use of levamisole as a cocaine adulterant [121].

If consumed by a pregnant female, all of the studied drugs might cross the placenta, some rapidly, as suggested by their placental clearance index (***CI***) relative to antipyrine. For some antiparasitic drugs (especially those from the pyrethroid family) investigated in this study, the placental permeability predicted using the ***CI*** values differs from predictions based on the fetus-to-mother concentration ratio (***FM***) reported earlier [94]. These differences imply excretion of these compounds via non-placental mechanisms or extensive metabolism, which requires detailed verification in the future.

The consequences of fetal exposure to veterinary antiparasitic drugs have not yet been fully recognized. Insecticides used to control ectoparasites in animals and/or humans, especially those from the chemical families of organophosphates, phenylpyrazoles (e.g., fipronil), pyrethroids, or carbamates, are toxic by design and, unfortunately, also affect off-target organisms. They are known or expected to disrupt human reproductive functions and pose a risk of developmental neurotoxicity [29,122]. Unfortunately, for most drugs in the studied group, there are no adequate data on their safety during pregnancy, which should prompt further research in this area.

In this study, we predicted interactions between antiparasitic drugs and a potential biological target in humans, namely acetylcholinesterase. Inhibiting AChE is currently one of the most studied approaches to slowing the progression of Alzheimer’s disease and improving cognitive function in patients with mild-to-moderate dementia [123]. However, prolonged, excessive exposure to AChE inhibitors is harmful, even for target patients [124,125]. As mentioned earlier, organophosphates irreversibly inhibit AChE by phosphorylating active-site serine, leading to prolonged enzyme inactivation and the accumulation of acetylcholine at synapses. Carbamates and urea derivatives (e.g., carbaryl, imidocarb, propoxur) temporarily inhibit AChE by transferring the carbamoyl group from the drug to a serine hydroxyl group in the enzyme’s active site [126,127]. Other antiparasitic drugs kill pests or impair their development through mechanisms other than AChE inhibition; however, reports suggest that AChE inhibition may be a secondary mechanism, for example, for some antiparasitic benzimidazoles [56,128]. Cypermethrin has been found to be a reversible AChE inhibitor in fish [129]. According to our analysis of the predicted AChE inhibition constants, almost all of the studied drugs might inhibit AChE, and their predicted ***K***_i_ values are comparable to those measured for registered AChE inhibitors reported in the CHEMBL database. Molecular docking studies suggested that the drugs might engage in several types of bonds/interactions with AChE, mainly with amino acids such as tryptophan (Trp86, Trp286), tyrosine (Tyr124, Tyr227, Tyr341), and phenylalanine (Phe295, Phe297, and Phe338), and with at least one amino acid from the AChE catalytic triad. However, because the molecular docking protocol used in this study was non-covalent, the results may be misleading for compounds that act through covalent mechanisms.

Hence, the antiparasitic drugs investigated in this study might cross the placenta, though incompletely in some cases. The majority of compounds in the studied group are either known or suspected AChE inhibitors, and further studies are needed to assess their effects on the placental and fetal cholinergic systems. Future investigations should include (i) placental and fetal metabolism and excretion of the drugs studied; (ii) mechanistic details of their binding to AChE (non-covalent vs. covalent); (iii) the stability of drug–enzyme complexes; (iv) separate analysis of the endpoints for salicylanilides, which (as mentioned earlier) are a distinct class of compounds whose unique features may not be fully captured by the models developed in this study. It should be emphasized that the modeled endpoints (placental ***CI***, AChE p***K***_i_) reflect transplacental transfer in an ex vivo perfusion system and the potential for AChE–drug interactions, not direct fetal toxicity. While transplacental transfer and inhibition of placental/fetal AChE might affect fetal development, the actual toxic effects depend on additional pharmacokinetic and pharmacodynamic factors beyond the scope of the present models.

## Figures and Tables

**Table 1:** Predicted ***CI*** and p***K***i values. Compounds highlighted in red fall outside the AD of the respective MLR models developed in this study, as indicated by Q-T^2^ analysis (Q and T^2^ above the respective thresholds). Compounds highlighted in yellow and underlined are untypical (elevated Q and “normal” T^2^).

No.	Compound	Family	*CI*_Eq(5)	p*K*_i__Eq(10)
A1	Thiabendazole	Benzimidazoles	0.83	6.18
A2	Parbendazole	Benzimidazoles	0.63	6.31
A3	Cambendazole	Benzimidazoles	0.69	6.23
A4	Albendazole	Benzimidazoles	0.61	5.82
A5	Fenbendazole	Benzimidazoles	0.62	5.75
A6	Flubendazole	Benzimidazoles	0.46	5.57
A7	Mebendazole	Benzimidazoles	0.55	5.51
A8	Oxfendazole	Benzimidazoles	0.51	5.19
A9	Oxibendazole	Benzimidazoles	0.62	6.20
A10	Luxabendazole	Benzimidazoles	0.42	4.86
A11	Febantel	Benzimidazoles	0.56	5.42
A12	Netobimin	Benzimidazoles	0.34	4.24
A13	Pyrantel	miscellaneous	0.86	6.84
A14	Morantel	miscellaneous	0.86	6.90
A15	Levamisol	miscellaneous	0.85	6.78
A16	Dichlorophos	organophosphate	0.71	5.81
A17	Haloxone	organophosphate	0.64	6.47
A18	Coumaphos	organophosphate	0.73	6.98
A19	Diethylcarbamazine	miscellaneous	0.84	6.91
A20	Bunamidine	miscellaneous	0.77	6.99
A21	Niclosamide	miscellaneous	0.49	4.92
A22	Dichlorophen	miscellaneous	0.72	5.75
A23	Bithionol	miscellaneous	0.68	5.04
A24	Diamfenetide	miscellaneous	0.51	5.24
A25	Triclabendazole	Benzimidazoles	0.72	5.75
A26	Oxiclosanide	Salicylanilide	0.53	4.62
A27	Brotianide	Salicylanilide	0.71	5.28
A28	Rafoxanide	Salicylanilide	0.64	5.53
A29	Clioxanide	Salicylanilide	0.70	5.59
A30	Closantel	Salicylanilide	0.68	5.64
A31	Nitroxinil	miscellaneous	0.53	**4.35**
A32	Niclofolan	miscellaneous	0.29	4.29
A33	Clorsulone	Sulfonamide	0.24	2.39
A34	Bromofenofos	organophosphate	0.38	**3.96**
A35	Epsiprantel	miscellaneous	0.82	8.77
A36	Resorantel	miscellaneous	0.47	4.32
A37	Praziquantel	miscellaneous	0.80	8.63
A38	Hexachlorophene	miscellaneous	0.66	4.98
A39	Sulfachloropyridazine	Sulfonamide	0.58	4.47
A40	Sulfadiazine	Sulfonamide	0.51	3.75
A41	Sulfadimethoxine	Sulfonamide	0.49	4.81
A42	Sulfadoxine	Sulfonamide	0.52	4.75
A43	Sulfamethoxazol	Sulfonamide	0.58	4.57
A44	Sulfadymidine	Sulfonamide	0.60	5.14
A45	Sulfaquinoxaline	Sulfonamide	0.53	4.96
A46	Trimethoprim	miscellaneous	0.58	4.69
A47	Pirymethamine	miscellaneous	0.70	5.31
A48	Diaveridine	miscellaneous	0.61	4.66
A49	Halofuginone	miscellaneous	0.37	5.33
A50	Arprinocide	miscellaneous	0.58	5.35
A51	Robenidine	miscellaneous	0.74	5.06
A52	Clopidol	miscellaneous	0.61	5.04
A53	Diclazuril	miscellaneous	0.53	5.01
A54	Clazuril	miscellaneous	0.51	4.92
A55	Toltrazuril	miscellaneous	0.51	5.80
A56	Dinitolmide	miscellaneous	0.34	4.21
A57	Aklomide	miscellaneous	0.56	4.74
A58	Diminazene	miscellaneous	0.25	1.77
A59	Pentamidine	miscellaneous	0.26	2.92
A60	Imidocarb	miscellaneous	0.57	5.11
A61	Amicarbalide	miscellaneous	0.14	1.46
A62	Diazinone	organophosphate	0.93	7.81
A63	cythioate	organophosphate	0.62	5.37
A64	fenthione	organophosphate	0.76	6.13
A65	Phoxim	organophosphate	0.93	6.61
A66	Trichlorfon	organophosphate	0.66	6.20
A67	Heptenophos	organophosphate	0.73	7.07
A68	Carbaryl	miscellaneous	0.83	6.28
A69	Propoxur	miscellaneous	0.81	6.29
A70	Allethrin	Pyrethroid	0.76	6.94
A71	Cypermethrin	Pyrethroid	0.70	6.14
A72	Flumethrin	Pyrethroid	0.65	6.37
A73	Cyfluthrin	Pyrethroid	0.64	6.21
A74	Cyhalothrin	Pyrethroid	0.64	5.90
A75	Permethrin	Pyrethroid	0.73	6.71
A76	Resmethrin	Pyrethroid	0.68	6.74
A77	Amitraz	miscellaneous	0.83	6.51
A78	Fipronil	miscellaneous	0.65	4.93
A79	Piperonyl butoxide	miscellaneous	0.65	7.55
A80	Diflubenzuron	miscellaneous	0.48	4.89
A81	Pyriproxifen	miscellaneous	0.81	6.84
A82	Methoprene	miscellaneous	0.72	6.54
A83	Lufenuron	miscellaneous	0.35	4.86
A84	Cyromazine	miscellaneous	0.69	4.73
A85	Tetramethrin	miscellaneous	0.66	7.94
A86	Rotenone	miscellaneous	0.65	7.69

## Data Availability

The data presented in this study are available in this manuscript.

## Supplementary Materials

The following supporting information can be downloaded at: https://www.mdpi.com/article/10.3390/ijms27135921/s1.

## References

[1] Tetro N., Moushaev S., Rubinchik-Stern M., Eyal S. (2018). The Placental Barrier: The Gate and the Fate in Drug Distribution. Pharm. Res..

[2] Rubinchik-Stern M., Eyal S. (2012). Drug Interactions at the Human Placenta: What Is the Evidence?. Front. Pharmacol..

[3] Blanco-Castañeda R., Galaviz-Hernández C., Souto P.C.S., Lima V.V., Giachini F.R., Escudero C., Damiano A.E., Barragán-Zúñiga L.J., Martínez-Aguilar G., Sosa-Macías M. (2020). The Role of Xenobiotic-Metabolizing Enzymes in the Placenta: A Growing Research Field. Expert Rev. Clin. Pharmacol..

[4] Mohammed A.M., Huuskonen P., Juvonen R., Sahlman H., Repo J., Myöhänen K., Myllynen P., Woo C.S.J., Karttunen V., Vähäkangas K. (2020). Activities of Metabolizing Enzymes in Human Placenta. Toxicol. Lett..

[5] Roe D.A., Little B.B., Bawdon R.E., Gilstrap L.C. (1990). Metabolism of Cocaine by Human Placentas: Implications for Fetal Exposure. Am. J. Obstet. Gynecol..

[6] Perić M., Bečeheli I., Čičin-Šain L., Desoye G., Štefulj J. (2022). Serotonin System in the Human Placenta—The Knowns and Unknowns. Front. Endocrinol..

[7] Silman I. (2021). The Multiple Biological Roles of the Cholinesterases. Prog. Biophys. Mol. Biol..

[8] Greenfield S.A., Zimmermann M., Bond C.E. (2008). Non-Hydrolytic Functions of Acetylcholinesterase: The Significance of C-Terminal Peptides. FEBS J..

[9] Wessler I., Kilbinger H., Bittinger F., Unger R., Kirkpatrick C.J. (2003). The Non-Neuronal Cholinergic System in Humans: Expression, Function and Pathophysiology. Life Sci..

[10] Lips K.S., Brü Ggmann D., Pfeil U., Vollerthun R., Grando S.A., Kummer W. (2005). Nicotinic Acetylcholine Receptors in Rat and Human Placenta. Placenta.

[11] Khosrow Tayebati S., Sabbatini M., Zaccheo D., Amenta F. (1997). Muscarinic Cholinergic Receptor Subtypes Expression by Human Placenta. Neurosci. Lett..

[12] Kitz R.J. (1964). The Chemistry of Anticholinesterase Activity. Acta Anaesthesiol. Scand..

[13] Colovic M.B., Krstic D., Lazarevic-Pasti T.D., Bondzic A., Vasic V.M. (2013). Acetylcholinesterase Inhibitors: Pharmacology and Toxicology. Curr. Neuropharmacol..

[14] Chrustek A., Hołyńska-Iwan I., Dziembowska I., Bogusiewicz J., Wróblewski M., Cwynar A., Olszewska-Słonina D. (2018). Current Research on the Safety of Pyrethroids Used as Insecticides. Medicina.

[15] Fukuto T.R. (1990). Mechanism of Action of Organophosphorus and Carbamate Insecticides. Environ. Health Perspect..

[16] Bajgar J., Kassa J., Fusek J., Kuca K., Jun D. (2015). Other Toxic Chemicals as Potential Chemical Warfare Agents. Handbook of Toxicology of Chemical Warfare Agents.

[17] Zhao Q., Yang G., Mei X., Yuan H., Ning J. (2009). Novel Acetylcholinesterase Inhibitors: Synthesis and Structure-Activity Relationships of Phthalimide Alkyloxyphenyl N,N-Dimethylcarbamate Derivatives. Pestic. Biochem. Physiol..

[18] Okuda T., Nomura Y., Konishi A., Misawa H. (2021). Competitive Inhibition of the High-Affinity Choline Transporter by Tetrahydropyrimidine Anthelmintics. Eur. J. Pharmacol..

[19] Sastry B.V.R. (1997). Human Placental Cholinergic System. Biochem. Pharmacol..

[20] González-García B., Olave M.E., Ramos-Martínez E., González-Horta C., Levario-Carrillo M., Sánchez-Ramírez B. (2008). Decrease of Muscarinic Cholinergic Receptors Expression in Placenta from Rats Exposed to Methyl Parathion. Hum. Exp. Toxicol..

[21] Mink P.J., Kimmel C.A., Li A.A. (2012). Potential Effects of Chlorpyrifos on Fetal Growth Outcomes: Implications for Risk Assessment. J. Toxicol. Environ. Health.

[22] Lassiter T.L., Padilla S., Mortensen S.R., Chanda S.M., Moser V.C., Barone S. (1998). Gestational Exposure to Chlorpyrifos: Apparent Protection of the Fetus?. Toxicol. Appl. Pharmacol..

[23] Ducci R.D.P., Kay C.S.K., Fustes O.J.H., Werneck L.C., Lorenzoni P.J., Scola R.H. (2021). Myasthenia Gravis during Pregnancy: What Care Should Be Taken?. Arq. Neuro-Psiquiatr..

[24] Harari R., Julvez J., Murata K., Barr D., Bellinger D.C., Debes F., Grandjean P. (2010). Neurobehavioral Deficits and Increased Blood Pressure in School-Age Children Prenatally Exposed to Pesticides. Environ. Health Perspect..

[25] Slotkin T.A. (2004). Cholinergic Systems in Brain Development and Disruption by Neurotoxicants: Nicotine, Environmental Tobacco Smoke, Organophosphates. Toxicol. Appl. Pharmacol..

[26] Furlong M.A., Herring A., Buckley J.P., Goldman B.D., Daniels J.L., Engel L.S., Wolff M.S., Chen J., Wetmur J., Boyd Barr D. (2017). Prenatal Exposure to Organophosphorus Pesticides and Childhood Neurodevelopmental Phenotypes. Environ. Res..

[27] Meyer A., Seidler F.J., Aldridge J.E., Slotkin T.A. (2005). Developmental Exposure to Terbutaline Alters Cell Signaling in Mature Rat Brain Regions and Augments the Effects of Subsequent Neonatal Exposure to the Organophosphorus Insecticide Chlorpyrifos. Toxicol. Appl. Pharmacol..

[28] Fish S.A. (1966). Organophosphorus Cholinesterase Inhibitors and Fetal Development. Am. J. Obstet. Gynecol..

[29] Abreu-Villaça Y., Levin E.D. (2017). Developmental Neurotoxicity of Succeeding Generations of Insecticides. Environ. Int..

[30] Uyar R., Turgut Y., Çelik H.T., Ünal M.A., Kuzukıran Ö., Özyüncü Ö., Ceylan A., Çinar Ö.Ö., Boztepe Ü.G., Özdağ H. (2024). Effects of DDT and DDE on Placental Cholinergic Receptors. Reprod. Toxicol..

[31] Byers D.M., Irwin L.N., Moss D.E., Sumaya I.C., Hohmann C.F. (2005). Prenatal Exposure to the Acetylcholinesterase Inhibitor Methanesulfonyl Fluoride Alters Forebrain Morphology and Gene Expression. Dev. Brain Res..

[32] Sharma A., Piplani P. (2013). Acetylcholinesterase Inhibitors from QSAR Point of View: How Close Are We?. Cent. Nerv. Syst. Agents Med. Chem..

[33] Wong K.Y., Duchowicz P.R., Mercader A.G., Castro E.A. (2012). QSAR Applications During Last Decade on Inhibitors of Acetylcholinesterase in Alzheimer’s Disease. Mini-Rev. Med. Chem..

[34] Vitorović-Todorović M.D., Cvijetić I.N., Juranić I.O., Drakulić B.J. (2012). The 3D-QSAR Study of 110 Diverse, Dual Binding, Acetylcholinesterase Inhibitors Based on Alignment Independent Descriptors (GRIND-2). The Effects of Conformation on Predictive Power and Interpretability of the Models. J. Mol. Graph. Model..

[35] Ansari F., Niazi A., Ghasemi J.B., Yazdanipour A. (2022). Docking and 2D-Structure-Activity Relationship and ADMET Studies of Acetylcholinesterase Inhibitors. Phys. Chem. Res..

[36] Recanatini M., Cavalli A., Hansch C. (1997). A Comparative QSAR Analysis of Acetylcholinesterase Inhibitors Currently Studied for the Treatment of Alzheimer’s Disease. Chem.-Biol. Interact..

[37] Recanatini M., Cavalli A., Belluti F., Piazzi L., Rampa A., Bisi A., Gobbi S., Valenti P., Andrisano V., Bartolini M. (2000). SAR of 9-Amino-1,2,3-4-Tetrahydroacridine-Based Acetylcholinesterase Inhibitors: Synthesis, Enzyme Inhibitory Activity, QSAR, and Structure-Based CoMFA of Tacrine Analogues. J. Med. Chem..

[38] Wong K.Y., Mercader A.G., Saavedra L.M., Honarparvar B., Romanelli G.P., Duchowicz P.R. (2014). QSAR Analysis on Tacrine-Related Acetylcholinesterase Inhibitors. J. Biomed. Sci..

[39] Castilho M.S., Guido R.V.C., Andricopulo A.D. (2007). 2D Quantitative Structure-Activity Relationship Studies on a Series of Cholesteryl Ester Transfer Protein Inhibitors. Bioorganic Med. Chem..

[40] Nekoeinia M., Yousefinejad S. (2021). QSAR Analysis of the Acetylcholinesterase Inhibitory Activity of Some Tertiary Amine Derivatives of Cinnamic Acid. Struct. Chem..

[41] Gao X., Tang J., Liu H., Liu L., Kang L., Chen W. (2018). Structure–Activity Relationship Investigation of Tertiary Amine Derivatives of Cinnamic Acid as Acetylcholinesterase and Butyrylcholinesterase Inhibitors: Compared with That of Phenylpropionic Acid, Sorbic Acid and Hexanoic Acid. J. Enzym. Inhib. Med. Chem..

[42] Nour H., Hashmi M.A., Belaidi S., Errougui A., El Kouali M., Talbi M., Chtita S. (2023). Design of Acetylcholinesterase Inhibitors as Promising Anti-Alzheimer’s Agents Based on QSAR, Molecular Docking, and Molecular Dynamics Studies of Liquiritigenin Derivatives. ChemistrySelect.

[43] Gao W., Ma X., Yang H., Luan Y., Ai H. (2022). Molecular Engineering and Activity Improvement of Acetylcholinesterase Inhibitors: Insights from 3D-QSAR, Docking, and Molecular Dynamics Simulation Studies. J. Mol. Graph. Model..

[44] Nour H., Abchir O., Belaidi S., Chtita S. (2022). Research of New Acetylcholinesterase Inhibitors Based on QSAR and Molecular Docking Studies of Benzene-Based Carbamate Derivatives. Struct. Chem..

[45] Khedraoui M., Karim E.M., Abchir O., Errougui A., Raouf Y.S., Samadi A., Chtita S. (2025). 2D-QSAR-Guided Design of Potent Carbamate-Based Inhibitors of Acetylcholinesterase. PLoS ONE.

[46] Pirmoradi S. (2023). Ligand-Based Pharmacophore Modeling to Identify Plant-Derived Acetylcholinesterase Inhibitor Natural Compounds in Alzheimer’s Disease. Jorjani Biomed. J..

[47] Ridzuan M.S.M., Jaafar M.Z., Zain M.M. (2012). Quantitative Structure-Activity Relationship (QSAR) Modelling of N-Aryl Derivatives as Cholinesterase Inhibitors. Proceedings of the 2012 IEEE Symposium on Humanities, Science and Engineering Research.

[48] Solomon K.A., Sundararajan S., Abirami V. (2009). QSAR Studies on N-Aryl Derivative Activity towards Alzheimer’s Disease. Molecules.

[49] Agbi J.I. (2025). Biophysical Approach of Modeling Inhibition Potency of Acetylcholinesterase for Possible Alzheimer Disease Treatment Using Genetic Algorithm Optimized Support Vector Regression. Int. J. Res. Publ. Rev..

[50] Wang Y., Zhao Y., Wei C., Tian N., Yan H. (2021). 4D-QSAR Molecular Modeling and Analysis of Flavonoid Derivatives as Acetylcholinesterase Inhibitors. Biol. Pharm. Bull..

[51] Gunda S.K., Pasula S., Gurram V., Shaik M. (2015). 3D QSAR and In Silico Docking Studies of Natural Flavonoid Derivatives as Acetylcholinesterase Inhibitors. Int. J. Pharm. Sci. Rev. Res..

[52] Chiou S.Y., Lai G.W., Tsai Y.H., Lin L.Y., Lin G. (2005). QSAR for Acetylcholinesterase and Butyrylcholinesterase Inhibition by Cardiovascular Drugs and Benzodiazepines. Med. Chem. Res..

[53] Alagoz M., Ozdemir Z., Zenni Y., Yilmaz T., Onkol T. (2020). QSAR and Pharmacophore Analysis on Pyridazinone Derivatives as Acetylcholinesterase Inhibitors. Ann. Med. Res..

[54] Khatabi K.E., Aanouz I., El-Mernissi R., Khaldan A., Ajana M.A., Bouachrine M., Lakhlifi T. (2020). 3d-Qsar and Molecular Docking Studies of p-Aminobenzoic Acid Derivatives to Explore the Features Requirements of Alzheimer Inhibitors. Orbital Electron. J. Chem..

[55] Tariq M., Hussain R., Sardar A., Rahim F., Khan S., Umer U., Rehman W., Khan Y., Iqbal T., Sarfraz H. (2025). Integrating Kinetic Mechanism of Novel Benzimidazole Derivatives as Potential Acetylcholinesterase Inhibitors: Insight into in-Vitro and in-Silico Approaches. J. Mol. Struct..

[56] El Khatabi K., El-Mernissi R., Aanouz I., Ajana M.A., Lakhlifi T., Shahinozzaman M., Bouachrine M. (2022). Benzimidazole Derivatives in Identifying Novel Acetylcholinesterase Inhibitors: A Combination of 3D-QSAR, Docking and Molecular Dynamics Simulation. Phys. Chem. Res..

[57] El Khatabi K., Aanouz I., El-Mernissi R., Singh A.K., Ajana M.A., Lakhlifi T., Kumar S., Bouachrine M. (2021). Integrated 3D-QSAR, Molecular Docking, and Molecular Dynamics Simulation Studies on 1,2,3-Triazole Based Derivatives for Designing New Acetylcholinesterase Inhibitors. Turk. J. Chem..

[58] El Khatabi K., Aanouz I., El-Mernissi R., Khaldan A., Ajana M.A., Bouachrine M., Lakhlifi T. (2020). Discovery of Novel Potential Anti-Alzheimer Hydrazones Derivatives Using 3D QSAR and Molecular Docking Studies. J. Mater. Environ. Sci..

[59] El Khatabi K., El-Mernissi R., Aanouz I., Ajana M.A., Lakhlifi T., Khan A., Wei D.Q., Bouachrine M. (2021). Identification of Novel Acetylcholinesterase Inhibitors through 3D-QSAR, Molecular Docking, and Molecular Dynamics Simulation Targeting Alzheimer’s Disease. J. Mol. Model..

[60] Pang X., Fu H., Yang S., Wang L., Liu A.L., Wu S., Du G.H. (2017). Evaluation of Novel Dual Acetyl- and Butyrylcholinesterase Inhibitors as Potential Anti-Alzheimer’s Disease Agents Using Pharmacophore, 3D-QSAR, and Molecular Docking Approaches. Molecules.

[61] Hammoudi N.E.H., Benguerba Y., Sobhi W. (2019). QSAR Modeling of Thirty Active Compounds for the Inhibition of the Acetylcholinesterase Enzyme. Curr. Res. Bioinform..

[62] Hammoudi N.-E.-H., Sobhi W., Attoui A., Lemaoui T., Erto A., Benguerba Y. (2021). In Silico Drug Discovery of Acetylcholinesterase and Butyrylcholinesterase Enzymes Inhibitors Based on Quantitative Structure-Activity Relationship (QSAR) and Drug-Likeness Evaluation. J. Mol. Struct..

[63] Ul-Haq Z., Mahmood U., Jehangir B. (2009). Ligand-Based 3D-QSAR Studies of Physostigmine Analogues as Acetylcholinesterase Inhibitors. Chem. Biol. Drug Des..

[64] Stellpflug S.J., Bangh S.A., Cole J.B. (2018). The Treatment of Maternal and Fetal Anticholinergic Toxicity with Physostigmine. Toxicol. Commun..

[65] Liu A.L., Guang H.M., Zhu L.Y., Du G.H., Lee S.M.Y., Wang Y.T. (2007). 3D-QSAR Analysis of a New Type of Acetylcholinesterase Inhibitors. Sci. China Ser. C Life Sci..

[66] Niu B., Zhao M., Su Q., Zhang M., Lv W., Chen Q., Chen F., Chu D., Du D., Zhang Y. (2017). 2D-SAR and 3D-QSAR Analyses for Acetylcholinesterase Inhibitors. Mol. Divers..

[67] Akula N., Lecanu L., Greeson J., Papadopoulos V. (2006). 3D QSAR Studies of AChE Inhibitors Based on Molecular Docking Scores and CoMFA. Bioorganic Med. Chem. Lett..

[68] Verma J., Khedkar V.M., Coutinho E.C. (2010). 3D-QSAR in Drug Design-A Review. Curr. Top. Med. Chem..

[69] Speck-Planche A., Luan F., Cordeiro M.N.D.S. (2012). Discovery of Anti-Alzheimer Agents: Current Ligand-Based Approaches toward the Design of Acetylcholinesterase Inhibitors. Rev. Med. Chem..

[70] Ghafouri H., Ranjbar M., Sakhteman A. (2017). 3D-QSAR Studies of Some Reversible Acetyl Cholinesterase Inhibitors Based on CoMFA and Ligand Protein Interaction Fingerprints Using PC-LS-SVM and PLS-LS-SVM. Comput. Biol. Chem..

[71] Muegge I., Mukherjee P. (2016). An Overview of Molecular Fingerprint Similarity Search in Virtual Screening. Expert Opin. Drug Discov..

[72] Yang J., Cai Y., Zhao K., Xie H., Chen X. (2022). Concepts and Applications of Chemical Fingerprint for Hit and Lead Screening. Drug Discov. Today.

[73] Bustamam A., Mushliha, Yanuar A., Anki P., Ulfa A. (2022). Evaluation Quantitative structure-Activity Relationship (QSAR) Using Ensemble Learning Methods on Acetylcholinesterase Inhibitors for Alzheimer’s Disease. Commun. Math. Biol. Neurosci..

[74] Simeon S., Anuwongcharoen N., Shoombuatong W., Malik A.A., Prachayasittikul V., Wikberg J.E.S., Nantasenamat C. (2016). Probing the Origins of Human Acetylcholinesterase Inhibition via QSAR Modeling and Molecular Docking. PeerJ.

[75] Lee S., Barron M.G. (2015). Development of 3D-QSAR Model for Acetylcholinesterase Inhibitors Using a Combination of Fingerprint, Molecular Docking, and Structure-Based Pharmacophore Approaches. Toxicol. Sci..

[76] Sobańska A.W., Sobański A.M., Brzezińska E. (2026). Antiparasitic Veterinary Drugs—In Silico Studies of Membrane Permeability, Distribution in the Environment, Human Oral Absorption and Transport Across the Blood–Brain Barrier. Membranes.

[77] Hewitt M., Madden J.C., Rowe P.H., Cronin M.T.D. (2007). Structure-Based Modelling in Reproductive Toxicology: (Q)SARs for the Placental Barrier. SAR QSAR Environ. Res..

[78] Lagrange F.J., Brun J.L., Clot P.F., Leng J.J., Saux M.C., Kieffer G., Bannwarth B.G. (2001). Placental Transfer of SR49059 in the Human Dually Perfused Cotyledon In Vitro. Placenta.

[79] Pacifici G.M. (2005). Transfer of Antivirals across the Human Placenta. Early Hum. Dev..

[80] Giaginis C., Zira A., Theocharis S., Tsantili-Kakoulidou A. (2009). Application of Quantitative Structure-Activity Relationships for Modeling Drug and Chemical Transport across the Human Placenta Barrier: A Multivariate Data Analysis Approach. J. Appl. Toxicol..

[81] Strani L., Cocchi M., Tanzilli D., Biancolillo A., Marini F., Vitale R. (2025). One Class Classification (Class Modelling): State of the Art and Perspectives. TrAC Trends Anal. Chem..

[82] Harnly J. (2025). One-Class Modeling for Verification of Botanical Identity: A Review. Front. Pharmacol..

[83] Alvarez-Ginarte Y.M., Crespo-Otero R., Marrero-Ponce Y., Noheda-Marin P., Garcia De La Vega J.M., Montero-Cabrera L.A., Ruiz García J.A., Caldera-Luzardo J.A., Alvarado Y.J. (2008). Chemometric and Chemoinformatic Analyses of Anabolic and Androgenic Activities of Testosterone and Dihydrotestosterone Analogues. Bioorganic Med. Chem..

[84] OECD (2007). OECD Environment Health and Safety Publications Series on Testing and Assessment No. 69 Guidance Document on the Validation of (Quantitative) Structure-Activity Relationship [(Q)SAR] MODELS.

[85] Dancis J., Kammerman S., Jansen V., Levitz M. (1983). The Effect of Ouabain on Placental Transport of 86Rb. Placenta.

[86] Dancis J., Lehanka J., Levitz M. (1985). Transfer of Riboflavin by the Perfused Human Placenta. Pediatr. Res..

[87] Rücker C., Rücker G., Meringer M. (2007). Y-Randomization and Its Variants in QSPR/QSAR. J. Chem. Inf. Model..

[88] Evotec Caco-2 Permeability Assay. https://www.evotec.com/solutions/drug-discovery-preclinical-development/cyprotex-adme-tox-solutions/adme-pk/drug-permeability-and-transporters/caco-2-permeability.

[89] Clark D.E. (1999). Rapid Calculation of Polar Molecular Surface Area and Its Application to the Prediction of Transport Phenomena. 2. Prediction of Blood-Brain Barrier Penetration. J. Pharm. Sci..

[90] Clark D.E. (1999). Rapid Calculation of Polar Molecular Surface Area and Its Application to the Prediction of Transport Phenomena. 1. Prediction of Intestinal Absorption. J. Pharm. Sci..

[91] Begum S., Jaswanthi P., Venkata Lakshmi B., Bharathi K. (2021). QSAR Studies on Indole-Azole Analogues Using DTC Tools; Imidazole Ring Is More Favorable for Aromatase Inhibition. J. Indian Chem. Soc..

[92] Canadian Society of Pharmacology and Therapeutics (CSPT) Inhibitory Constant (Ki). https://pharmacologycanada.org/Inhibitory-constant-ki.

[93] Reynoso-García M.F., Nicolás-Álvarez D.E., Tenorio-Barajas A.Y., Reyes-Chaparro A. (2025). Structural Bioinformatics Applied to Acetylcholinesterase Enzyme Inhibition. Int. J. Mol. Sci..

[94] Sobańska A.W. (2023). In Silico Assessment of Risks Associated with Pesticides Exposure during Pregnancy. Chemosphere.

[95] Zhang Y., Kua J., McCammon J.A. (2002). Role of the Catalytic Triad and Oxyanion Hole in Acetylcholinesterase Catalysis: An Ab Initio QM/MM Study. J. Am. Chem. Soc..

[96] Zhou Y., Wang S., Zhang Y. (2010). Catalytic Reaction Mechanism of Acetylcholinesterase Determined by Born-Oppenheimer Ab Initio QM/MM Molecular Dynamics Simulations. J. Phys. Chem. B.

[97] Johnson G., Moore S. (2006). The Peripheral Anionic Site of Acetylcholinesterase: Structure, Functions and Potential Role in Rational Drug Design. CPD.

[98] Hung L.W., Sanbonmatsu K.Y., Williams R.F., Chen J.C.H. (2025). Acetylcholinesterase: Structure, Dynamics, and Interactions with Organophosphorus Compounds. Protein Sci..

[99] Gaulton A., Hersey A., Nowotka M.L., Patricia Bento A., Chambers J., Mendez D., Mutowo P., Atkinson F., Bellis L.J., Cibrian-Uhalte E. (2017). The ChEMBL Database in 2017. Nucleic Acids Res..

[100] Gaulton A., Bellis L.J., Bento A.P., Chambers J., Davies M., Hersey A., Light Y., McGlinchey S., Michalovich D., Al-Lazikani B. (2012). ChEMBL: A Large-Scale Bioactivity Database for Drug Discovery. Nucleic Acids Res..

[101] Butini S., Guarino E., Campiani G., Brindisi M., Coccone S.S., Fiorini I., Novellino E., Belinskaya T., Saxena A., Gemma S. (2008). Tacrine Based Human Cholinesterase Inhibitors: Synthesis of Peptidic-Tethered Derivatives and Their Effect on Potency and Selectivity. Bioorganic Med. Chem. Lett..

[102] Hamulakova S., Janovec L., Hrabinova M., Kristian P., Kuca K., Banasova M., Imrich J. (2012). Synthesis, Design and Biological Evaluation of Novel Highly Potent Tacrine Congeners for the Treatment of Alzheimer’s Disease. Eur. J. Med. Chem..

[103] Lin G., Tseng H.-C., Chio A.-C., Tseng T.-M., Tsai B.-Y. (2005). A Rate Determining Step Change in the Pre-Steady State of Acetylcholinesterase Inhibitions by 1,*n*-Alkane-Di-*N*-Butylcarbamates. Bioorganic Med. Chem. Lett..

[104] Zilbeyaz K., Oztekin A., Kutluana E.G. (2021). Design and Synthesis of Garlic-Related Unsymmetrical Thiosulfonates as Potential Alzheimer’s Disease Therapeutics: In Vitro and In Silico Study. Bioorganic Med. Chem..

[105] Lopes J.P.B., Silva L., Lüdtke D.S. (2021). An Overview on the Synthesis of Carbohydrate-Based Molecules with Biological Activity Related to Neurodegenerative Diseases. RSC Med. Chem..

[106] Butini S., Campiani G., Borriello M., Gemma S., Panico A., Persico M., Catalanotti B., Ros S., Brindisi M., Agnusdei M. (2008). Exploiting Protein Fluctuations at the Active-Site Gorge of Human Cholinesterases: Further Optimization of the Design Strategy to Develop Extremely Potent Inhibitors. J. Med. Chem..

[107] Sun Q., Peng D.-Y., Yang S.-G., Zhu X.-L., Yang W.-C., Yang G.-F. (2014). Syntheses of Coumarin–Tacrine Hybrids as Dual-Site Acetylcholinesterase Inhibitors and Their Activity against Butylcholinesterase, Aβ Aggregation, and β-Secretase. Bioorganic Med. Chem..

[108] Fu L., Shi S., Yi J., Wang N., He Y., Wu Z., Peng J., Deng Y., Wang W., Wu C. (2024). ADMETlab 3.0: An Updated Comprehensive Online ADMET Prediction Platform Enhanced with Broader Coverage, Improved Performance, API Functionality and Decision Support. Nucleic Acids Res..

[109] Moriwaki H., Tian Y.S., Kawashita N., Takagi T. (2018). Mordred: A Molecular Descriptor Calculator. J. Cheminform..

[110] Sushko I., Novotarskyi S., Körner R., Pandey A.K., Rupp M., Teetz W., Brandmaier S., Abdelaziz A., Prokopenko V.V., Tanchuk V.Y. (2011). Online Chemical Modeling Environment (OCHEM): Web Platform for Data Storage, Model Development and Publishing of Chemical Information. J. Comput.-Aided Mol. Des..

[111] Vainio M.J., Johnson M.S. (2007). Generating Conformer Ensembles Using a Multiobjective Genetic Algorithm. J. Chem. Inf. Model..

[112] Gallagher N.B., O’Sullivan D. Selection of Representative Learning and Test Sets Using the Onion Method. https://eigenvector.com/wp-content/uploads/2022/10/Onion_SampleSelection.pdf.

[113] Daoud J.I. (2018). Multicollinearity and Regression Analysis. J. Phys. Conf. Ser..

[114] Gadaleta D., Mangiatordi G.F., Catto M., Carotti A., Nicolotti O. (2016). Applicability Domain for QSAR Models. Int. J. Quant. Struct. Prop. Relatsh..

[115] Roy K., Kar S., Ambure P. (2015). On a Simple Approach for Determining Applicability Domain of QSAR Models. Chemom. Intell. Lab. Syst..

[116] Sahigara F., Mansouri K., Ballabio D., Mauri A., Consonni V., Todeschini R. (2012). Comparison of Different Approaches to Define the Applicability Domain of QSAR Models. Molecules.

[117] Weaver S., Gleeson M.P. (2008). The Importance of the Domain of Applicability in QSAR Modeling. J. Mol. Graph. Model..

[118] Gissi A., Gadaleta D., Floris M., Olla S., Carotti A., Novellino E., Benfenati E., Nicolotti O. (2014). An Alternative QSAR-Based Approach for Predicting the Bioconcentration Factor for Regulatory Purposes. Altex.

[119] Kochnev Y., Ahmed M., Maldonado A.M., Durrant J.D. (2024). MolModa: Accessible and Secure Molecular Docking in a Web Browser. Nucleic Acids Res..

[120] O’Boyle N.M., Banck M., James C.A., Morley C., Vandermeersch T., Hutchison G.R. (2011). Open Babel: An Open Chemical Toolbox. J. Cheminform..

[121] Chang A., Osterloh J., Thomas J. (2010). Levamisole: A Dangerous New Cocaine Adulterant. Clin. Pharmacol. Ther..

[122] Calderón-Garcidueñas A.L., Martínez-Valenzuela M.d.C., Waliszewski-Kubiak S.M. (2023). Pesticide Exposure and Its Effects on Intrauterine and Postnatal Development. Rev. Perinatol. Y Reprod. Humana.

[123] Moss D.E. (2020). Improving Anti-Neurodegenerative Benefits of Acetylcholinesterase Inhibitors in Alzheimer’s Disease: Are Irreversible Inhibitors the Future?. Int. J. Mol. Sci..

[124] Bittner N., Funk C.S.M., Schmidt A., Bermpohl F., Brandl E.J., Algharably E.E.A., Kreutz R., Riemer T.G. (2023). Psychiatric Adverse Events of Acetylcholinesterase Inhibitors in Alzheimer’s Disease and Parkinson’s Dementia: Systematic Review and Meta-Analysis. Drugs Aging.

[125] Schneider L.S. (2012). Could Cholinesterase Inhibitors Be Harmful over the Long Term?. Int. Psychogeriatr..

[126] Darvesh S., Darvesh K.V., McDonald R.S., Mataija D., Walsh R., Mothana S., Lockridge O., Martin E. (2008). Carbamates with Differential Mechanism of Inhibition toward Acetylcholinesterase and Butyrylcholinesterase. J. Med. Chem..

[127] Yadav C.S., Kumar V., Suke S.G., Ahmed R.S., Mediratta P.K., Banerjee B.D. (2010). Propoxur-Induced Acetylcholine Esterase Inhibition and Impairment of Cognitive Function: Attenuation by Withania Somnifera. Indian J. Biochem. Biophys..

[128] Türkan F. (2021). Investigation of the Toxicological and Inhibitory Effects of Some Benzimidazole Agents on Acetylcholinesterase and Butyrylcholinesterase Enzymes. Arch. Physiol. Biochem..

[129] Ahmed O., Mastan S.A., Banu S.R., Indira P. (2015). Sub-Lethal Effect of Cypermethrin on Acetylcholinesterase (AChE) Activity and Acetylcholine (Ach) Content in Selected Tissues of *Channa striatus* (Bloch.). J. Toxicol. Environ. Health Sci..

